# Impact of early life shocks on educational pursuits–Does a fade out co-exist with persistence?

**DOI:** 10.1371/journal.pone.0275871

**Published:** 2022-10-13

**Authors:** Gaurav Dhamija, Gitanjali Sen

**Affiliations:** 1 Indian Institute of Technology Hyderabad, Telangana, India; 2 Department of Economics, Shiv Nadar Institution of Eminence Deemed to be University, Uttar Pradesh, India; University of Luxembourg and Luxembourg Institute of Socio-Economic Research (LISER), LUXEMBOURG

## Abstract

**Background:**

Changes in climatic conditions have increased the variability in rainfall patterns worldwide. A negative rainfall shock faced by children in the initial 1000 days of life and the resulting malnutrition can harm the likelihood of children’s survival, overall growth, development of the brain, motor skills, and cognitive abilities, leading to poor performance in education and labor market. While the existing findings about the long-run outcomes are mixed, it is essential to understand the nuances in such an estimation.

**Methods:**

Using the exogenous variation in rainfall in India, we estimate the impact of adverse shocks at birth on the cognitive abilities of children at ages 5, 8, 12, and 15, on educational attainments, and the likelihood of studying STEM at higher secondary school.

**Results:**

The Young Lives Survey data from Andhra Pradesh, India, presents evidence of the negative impact of rainfall shocks at birth on cognitive abilities from age 5 to 8, attenuating at age 12. Using nationally representative data, while we investigate the impact of adverse rainfall shocks at birth on academic performance measured by the high school grades and STEM choice at higher secondary school, we do not find a persistent impact.

**Conclusion:**

We unfold the impact of rainfall shocks on a chain of outcomes connected to long-run educational pursuits, as it helps to identify the most crucial stage for policymaking. Since STEM subjects are strongly associated with the labor market, connecting the association with early life shocks seems to be an essential addition to the literature. While we find evidence of reduced cognitive abilities in the early years, those do not seem to persist in the long run. The potential sample selection or attrition biases and the estimates of those biases can explain the nuances of estimating the long-run impact of adverse shocks at birth.

## 1 Introduction

The “fetal origin theory” [1] suggests that the conditions during the *in-utero* period have significant effects on later-life health outcomes because poor nutrition during the crucial periods of gestation affects the growth of the fetus. Children who face malnutrition in-utero or in the initial 1000 days of their life can suffer from immediate effects [2] like the reduced likelihood of survival, lesser growth and development, and lasting impact on their long-term health conditions [3–7]. It also includes poor physical growth of the brain leading to poor development of motor skills and reduced motivation level–all of that may be responsible for the adverse effects of malnutrition on cognitive achievement. As the childhood is a crucial period for the development of cognitive abilities [8, 9], it is more likely that the poor health status of the cohort of children causes delays in school enrollment, lower attendance rates, results in poor test scores and lesser years of education [10–13].

While the mortality bias is difficult to estimate as we do not observe the children who do not survive, the findings from the existing literature estimating the impacts of such early life adverse shocks on education outcomes of surviving children are mixed. The long-lasting impact of adverse shocks in-utero or childhood is the net outcome of two opposite effects [14] among the surviving children. Firstly, it brings malnutrition in the affected children. This may affect the growth and development of the body in such a way that the children underperform in later lives. Secondly, while adverse shocks reduce the likelihood of survival of the affected children, the surviving children tend to acquire or have the unobserved additional capability, which makes them stronger than their unaffected counterparts. The net effect is what the researchers end up estimating in the long run, where a negative effect means the former channel dominates the latter. The empirical challenges of finding an answer to which of these effects dominates among the surviving children primarily emanate from the facts that 1) acquired ability is difficult to measure, and 2) the parental investments following a negative shock are endogenous. The parental investments and human development outcomes are jointly determined by parental preferences and resources [15], making identification a considerable challenge.

Therefore, while interpreting the impact on long-run education outcomes, the primary challenge is that we do not have a direct mechanism of estimating the effects on the education of surviving children compared to the children who do not make up to that level. This leads to an estimation of the impact on the surviving children only. Such estimates tend to suffer from attrition bias. Attrition could arise due to mortality or due to sample attrition. Additionally, even among surviving children, continuing education at each level involves a decision, leading to a typical problem of sample selection bias.

The objective of this paper is to highlight the nuances in estimating the impact of early life shock on later life education outcomes and disentangle the root of mixed findings available in the existing literature. In the process, we investigate what happens throughout the life course of a child’s educational pursuits when she faces negative shocks in her early life. Therefore, among the surviving children, we estimate the effects at each stage of the education ladder to see if a negative effect is found in early life, and if so, how long the negative effect persists, and at what point the effect dissolves, if at all. In this way, instead of measuring only a long-run outcome on surviving children, we are able to analyze outcomes over a period of time. We discuss a few ways of addressing the potential biases due to mortality selection, sample attrition or sample selection, and present a few additional estimates to address these concerns. Our estimates, supported by a series of tests for robustness, along with the analysis of heterogeneous effects are expected to guide researchers on the nuances of measuring the long-run impact of early life shocks.

Since rainfall shocks in early childhood can provide an exogenous source of malnutrition potentially affecting the cognitive abilities of the children [16], using the information on rainfall shocks during the birth year of the children, we estimate the impacts on a series of outcomes connected to educational pursuits. The outcomes considered are cognitive development at different stages of childhood, completion of education at different levels, grades in secondary school examination, and subject choice at the higher secondary level (Grades in this study imply division awarded after the completion of 10^th^ standard i.e., 1^st^, 2^nd^, and 3^rd^ division, with 1^st^ being the best performance). These estimates are not only expected to disentangle at what stages we are able to identify a stronger negative impact, but increased efforts targeted to those specific stages can also help in efficient policymaking. Should targeting the cognitive development of the affected children early in life be the primary policy recommendation, or should we worry equally about participation and grades at each stage of education, leading up to the subject choice in higher education because the latter is connected to labor market outcomes [17]? We attempt to unfold the chain of outcomes, which can be a guide to policymakers for identifying the most crucial stage. We also test the robustness of our estimates of parental investment in the education of school-going children to check if any reinforcing investment by parents is able to attenuate the negative effects of early life cognitive abilities. The negative effects on cognitive abilities seem to be too strong to be attenuated by such reinforcing investments. However, the negative effects do not seem to persist in later years.

Changes in climatic conditions have increased the variability in rainfall patterns throughout the world in the last few decades. This has been a serious concern to the countries where most of the population stays in the rural areas with agriculture being their primary source of livelihood. The vulnerability to weather shocks like drought has direct and indirect effects on families from multiple dimensions, including long-lasting impacts on the welfare of the children [18–23]. This question warrants particular importance in the context of India, where, about 66.5 percent of males and 83.3 percent of females are reported to have agriculture as their principal economic activity [24]. Some areas of the country face shortage in rainfall every year, which leads to drought-like situations, thereby, negatively affecting the agricultural output and the income of the 73 percent of the rural population engaged in agriculture [25]. It leads to a reduction in the consumption levels of children at an early age, affecting their overall growth [16]. Since India is home to about 50 percent of the world’s undernourished children [26], it qualifies to be the most suitable sample for our analysis.

While over the last few years, a large body of literature has emerged that looks at the impact of various natural shocks such as floods, epidemics, droughts, and famine [14, 27–29], none of the studies specifically explores the impact on a sequence of outcomes from the early stage of cognitive development, to the educational pursuits at schools, up to the subject choice in higher secondary school. Unless one is able to estimate the impact on a chain of education outcomes from very early life till later life, it is difficult to disentangle the stages when children are worst affected, and when the net impact is null. Without such a thorough analyses of a chain of outcomes, we tend to generalize the interpretation of a null impact on long-run education outcomes. Identifying the worst affected stages of the children, while disentangling the sources of mixed impacts on long-run education pursuits, is our contribution to the literature. We supplement our findings through additional analysis of potential problems related to attrition bias or selection bias that are important in studies of long-run outcomes. Shah and Steinberg [16], in their study also use early life rainfall shocks and find that positive rainfall shock in early childhood improves test scores, and increases the likelihood of enrollment in school, and age-relevant grades in the school. However, we focus on the negative rainfall shocks and their effects throughout different stages till the post-secondary subject choice.

Overall, we find a negative impact of rainfall shocks at birth on cognitive abilities in early life, but do not find any evidence of sustained impact in later life. Using the Young Lives Survey (YLS) data, we observe that the negative impact persists from age 5 to age 8. Then it attenuates from age 12. We do not observe any effect on cognitive ability at age 12 and 15. After further investigations of impacts on academic performance measured by the high school grades, and on the selection of STEM (Science, Technology, Engineering, and Mathematics) as a subject choice at the higher secondary level, we do not seem to find any impact while using the India Human Development Survey-2 [30] data. Our explanation for this result is twofold: One, the effect on cognitive abilities in early life is most crucial and the effect may dilute in later life due to the unobserved ability of the survivors (mortality bias). Two, while measuring educational outcomes in later life, we do not observe the children who may have dropped out early in life due to the adverse shocks and we only observe the better ones (selection bias or sample attrition bias). The combination of the above may cause a null impact on later life outcomes. This is evident from the fact that the likelihood of completion of school till the secondary level is lower for the affected cohort. Does the selected (observed) sample increase likelihood of dropping out of the weaker children, thereby suppressing the adverse effects?

Lee bounds are expected to address such issues when reasons for sample attrition are due to mortality selection, non-random survey response, or our inability to follow observations over time [31]. It provides the worst- and best-case scenarios for the missing outcomes using the observed data, which, in our case corroborates the fact that sample attrition at the education outcomes of the STEM choice could be the reason for seemingly null impact in the long run. This argument is strengthened by the fact that the Lee bounds at the lower end are found to be significantly negative in a few cases when the overall effect seems insignificant.

This is an interesting finding for the policymakers because it offers a few explanations for the mixed impact of adverse shocks on the educational outcomes of children as observed in the literature (see Shah and Steinberg [16] for a discussion on this). Our paper is likely to add to the strand of literature that attempts to estimate the impact of early life shocks on educational pursuits in later life. As we move to the later stages, we lose out on the most critical period of the child’s life course and there may not be any opportunity to catch up if the children drop out of the education system.

Our finding is in congruence with the earlier studies of Stein et al. [32] and Villar et al. [33], where they did not find any effect of in-utero shock from famine on the cognitive development of children in their adulthood. Nübler et. al. [34] estimate the impact of rainfall shocks received in utero, or separately at each age till the first seven years of life, on cognitive development, the likelihood of school enrollment, and total years of schooling completed among 11–14 years old girls of Kenya. They too find significant effects of early life rainfall shocks on the likelihood of school enrollment, total years of schooling, and mathematics and language test scores of 11–14 years old girls. This effect is limited only when shocks are received in the first 2–3 years of life, including in-utero, while the shocks received in later years do not seem to have significant adverse effects. However, they do not measure the impact of shocks on outcomes beyond 14 years of age, making it difficult to conclude whether the effects of shocks persist till adulthood and beyond. They find that shocks received at ages 1, 3, 4, and 7 are associated with lower expectations of girls about completing primary school. Early life shocks are found to have negative health impacts and persistent effects on cognitive scores, which are argued to be the mechanisms behind lower schooling. They also find an unconditional cash transfer program to have mitigating effects in early life.

Adhvaryu et al. [35] find that adverse rainfall shocks in the year of birth reduce grade attainment, post-secondary school enrollment, and employment outcomes in Mexico. Using one of the most extensive cash incentive programs, Progresa, they find that recovering from early life shock is possible. While their paper helps find the channels for mitigation, our objective is not targeted to any specific age. We plan to find a justification for the mixed impact as found in the literature.

The work closest to ours is the one by Chang et al. [36], who use the Young Lives data to study the effects of rainfall shock on cognitive and non-cognitive outcomes of children in the state of Andhra Pradesh, India. They measure the impact of in-utero rainfall shocks on cognitive outcomes at different ages, and a few non-cognitive outcomes like an individual’s sense of agency and self-esteem. On cognitive outcomes, like our findings, they too find that the negative effects of shocks are more pronounced at age 5 and not significant in later years. However, our study is an extension from a few aspects. One, unlike them, we do not restrict our study to only a single state of India. Instead, we supplement that sample with a nationally representative sample (IHDS-2), which provides an external validity. Two, we study the effects of shocks up to high school subject choice, which is an addition to the literature. Since the choice of STEM as a subject is highly connected to the labor market and is high in demand in India [17], connecting the association with early life shock seems to be a natural choice and interesting addition to the literature too. Three, Chang et al. [36] do not analyze the reasons for seemingly null effects in later life, which seems to be a significant contribution by us. Using Lee bounds, we are able to provide supporting evidence for our arguments for the attenuated effects in later life.

In the next section, we explain the methodology and data. Section 3 explains the main results. A few potential concerns have been discussed in section 4, and we conclude in section 5 with potential policy recommendations.

## 2 Materials and methods

### 2.1 Contextual framework

Indian school system can be divided into four levels, with standards (or class of study) I-V being primary, standards VI-VIII being elementary or middle, standards IX-X being secondary, and XI-XII being the higher secondary level. Primary education in government schools does not involve direct costs, such as cost of tuition fees, books, and uniforms. But due to concerns regarding the quality of government schools, a significant section of the population opts for private schools. From 2009, due to the implementation of the Right to Education Act, and therefore during the years of the surveys used in this paper, middle school education has also been made free of tuition fees in government schools. However, tuition fees for secondary and higher secondary education must be borne by the users, even in government schools. Students need to write exams conducted by the respective state boards or central board of education at the end of secondary level and higher secondary levels. These are high-stake large-scale examinations where no school has any control over the examinations or evaluations conducted by the boards. The grades received at the secondary level gain considerable importance while opting for STEM (Science, Technology, Engineering, and Mathematics) subjects at the higher secondary level because usually schools require students to have certain minimum grades at the secondary level. However, this decision is decentralized at the school level, and the examination boards do not have any role in the subject choice. Since all Indian schools do not have higher secondary classes, students may have to change schools after the secondary level, and the grade received at the secondary level gains more importance in that case. An OLS estimation (using IHDS-2 data, see appendix section B4) reveals that there is a 20-percentage point higher likelihood of opting for STEM at the higher secondary level when the student is awarded first division at the secondary level, as compared to the ones being awarded second or third division.

### 2.2 Data

#### Household survey data from the YLS

We use the YLS data for estimating early life outcomes such as cognitive development at different stages. YLS is a longitudinal cohort study of children designed to examine the drivers of childhood poverty in Ethiopia, India, Peru, and Vietnam, where the Indian sample covers only the state of Andhra Pradesh. For our analysis, we focus on school-aged children from 2002 to 2017 in Andhra Pradesh [37], India, spread over five waves. At the start of the survey in 2002, Andhra Pradesh had 23 administrative districts that were further subdivided into 1,125 mandals and 27,000 villages. The survey is conducted in the city of Hyderabad and the districts of Anantapur, YSR Kadapa, Srikakulam, West Godavari, Karimnagar, and Mahbubnagar (Karimnagar and Mahbubnagar are now a part of a separate state Telangana). Andhra Pradesh has been bifurcated into two states named Andhra Pradesh and Telangana in June 2014. These seven study points were used to cover 100 communities spread across 20 sentinel sites, where a sentinel site is defined as equivalent to an administrative sub-district (Mandal). These details on the survey methodology are reported in Kumra [38].

YLS accumulated extensive information on 2,011 children aged between 6 to 21 months (the Younger Cohort) and 1,008 children aged between 7.5 to 8.5 years (the Older Cohort born in 1994 and 1995) for the first survey round in the year 2002. The comprehensive survey of the Young Lives children and their primary caregivers was subsequently conducted in the years 2006–07, 2009–10, 2013–14, and 2016–17. Our analysis uses data from rounds 2 to 5 for the younger cohort, when children were aged around 5, 8, 12, and 15 years. We exclude the first (2002) round of data as no test was conducted to measure the cognitive ability of the children in this round. We limit our analysis to the younger cohort as the children in the older cohort were already 12 years old by the second round. Moreover, there is no way to estimate cognitive ability at the young age of 5 and 8 years.

Our primary outcome variables from the YLS data are the Peabody Picture Vocabulary Test (PPVT) and mathematics test scores at the age of 5, 8, 12, and 15 years. For both the PPVT and the mathematics tests, we convert the raw test scores to Item Response Theory (IRT) test scores. The latter convert all raw test scores into z-scores, so that interpretation can be done in standard deviation units. This method has been used by Singh [39]. IRT models help in accounting for the difficulty in the questions (for more details on IRT models see Das and Zajonc, [40]; Van der Linden and Hambleton, [41]).

We use gender, father’s education (whether the child’s father has attained formal education or not), mother’s education (whether the child’s mother has attained formal education or not), mother’s height (in cm), number of family members in the household, religion (whether the child belongs to Hindu, Muslim, or other religion), ethnicity (whether the child belongs to Schedule Castes abbreviated as SC, Schedule Tribes abbreviated as ST, Other Backward Classes abbreviated as OBC, or any other Castes), wealth status (five quantiles, indicating the poorest, poor, middle, rich or richest group, created from the wealth index provided by the YLS), and district level dummies as our additional covariates.

Our analytical sample consists of 1264 children belonging to the younger cohort across every round from 2 to 5. These are the children living in rural areas with non-missing and valid information for the outcome variables, independent variables of interest, and covariates. Fig A1 in S1 Appendix presents a flow chart explaining the sample attrition caused by missing information on the variables, which is about 12.8%-15.9% of our overall sample.

#### Household survey data from the IHDS-2

For estimating the impacts on later life outcomes, such as the likelihoods of completion of different levels of schooling, grades (capturing indicators of performances) in secondary school examination, and choice of STEM at the higher secondary school, we use the publicly available second round of the IHDS data. This data is collected jointly by the University of Maryland and the National Council of Applied Economic Research (NCAER), New Delhi during the years 2011–12. It is a nationally representative multi-topic panel survey covering 42,152 households in 1,420 villages and 1,042 urban blocks across 384 districts, spread over 33 states and union territories in India. It covers all the states and union territories of India except the Andaman and Nicobar, and the Lakshadweep. This survey captures information related to health, education, employment, economic status, marriage, fertility, gender relations, and social capital of the household members. There are two waves of this nationally representative dataset. The first wave collected data from 41,554 households in 2004–05. In the second wave (2011–12), 83 percent of these households were re-interviewed along with an additional sample of 2,134 households. However, we use the second wave (IHDS-2) for our analysis.

Detailed information about the education of each member of the household surveyed is available in the education section of the Income and Social Capital Questionnaire. It records the years of education completed by all the members of the household. Individuals who never enrolled in school are reported to complete zero years of education. All individuals completing ten years of schooling are also asked questions about the subject they opted for at the higher secondary level. Their answers about their courses of study are available in the following categories (see appendix B3): 1) Arts, 2) Commerce, 3) Science, 4) Engineering, 5) Agriculture 6) Home Science/Craft/Design, 7) Other Technology/ Vocational, and 8) Others.

Therefore, the analyses of our IHDS-2 sample begin with 35,926 individuals in the age group of 11–40 years for the outcome of completion of primary schooling. The sample gets further limited to 6,817 individuals in the age group of 15–40 years, who report their subject choice in the higher secondary school. The summary statistics presented in Table 2 list the sample size for each of our outcomes of interest. These are the children from households whose income source is cultivation or allied agriculture or agriculture wage labor, living in rural areas with non-missing and valid information for the outcome variables, independent variable of interest, and other covariates.

Since the typical age at which students are expected to enter standard I of the formal school is six years with an expected duration of completion being one year, we restrict the minimum age to 11, 14, and 16 years for the analysis of completion of primary, middle and secondary level of schooling, respectively. Moreover, we keep the minimum age to 15 years for the analysis of grades awarded in the 10^th^ standard and for the subject choice, because students get to enroll in standard XI at the age of 15 or 16 years on average.

#### Rainfall data from the University of Delaware

Our primary predictor variable is the *negative rainfall shock* in the birth year of the individuals from the younger cohort (born in 2001 and 2002) of the YLS data or of the individuals from the IHDS-2 data. To construct the rainfall shock years at the district level, we use monthly rainfall data collected from the University of Delaware [42]. It is gridded by the longitude and latitude lines and spans all of India from 1900 to 2014. In this paper, we use this data from 1970 to 2012. We merge this district-level monthly rainfall data with the districts of YLS and IHDS-2 data.

To locate the level of rainfall of the surveyed district for every year, we match the gridded lines with the nearest point on the grid to the center of the district. We calculate rainfall for any birth year in a district as the sum of rainfall in all 12 months for that specific year in that district. In addition, we find the mean of annual rainfall in a specific district by using the annual rainfall data from 1970 to 2012 for all the birth years. Mean annual precipitation for a particular birth year in a district excludes the rainfall of that year in the district. Using the strategy of Maccini and Yang [21], we calculate the deviation of the natural log of birth year rainfall and the natural log of mean annual rainfall in the given district. We define rainfall shock year as a binary variable that takes a value of 1, if the deviation of the natural log of birth year rainfall from the natural log of mean annual rainfall in that year of a district is less than 0, otherwise, it assumes a value of 0. The frequency density of children exposed to rainfall deviations from the long-term average rainfall is presented Fig A2 in S1 Appendix.

### 2.3 Methods: Identification

#### Ethics statement

We use three different data sets for this work from the publicly available data portal, as cited in the reference section and explained earlier. We do not conduct any primary surveys. All three data sets have been made available to researchers on request, and the data were analyzed anonymously. Hence, we did not require any ethical consent.

#### Identification

Assuming an exposure to the rainfall shock to be random, we estimate the following specification using the OLS method. Our identification strategy is based on assumptions of random spatial and temporal variation in rainfall:

$$Y_{ithd}=\beta_{0}+\beta_{1}({Shockinbirthyear}_{dt})+\alpha_{t}+{\gamma Male}_{i}+{\theta H}_{h}+\delta_{d}+\varepsilon_{ithd}$$ (1)

Here, the early-life rainfall shock in the birth year, as explained earlier, is captured by the binary variable *Shockinbirthyear*_*dt*_, (= 1 indicates shock).

The outcome variable ‘Y_*ithd*_*’* considered in alternate specifications for an individual ‘*i*,’ belonging to cohort *‘t’* from household ‘*h*,’ of, district ‘*d*,’ are:

i) Eight measures capturing the cognitive development of the child till 15 years of age. That are, the PPVT scores at the age 5, 8, 12, and 15, Cognitive Development Assessment (CDA) score at the age 5 (see appendix B2), and Mathematics Achievement Test (MAT) scores at the age of 8, 12 and 15. We use YLS data for this part of the analysis due to the availability of detailed outcomes at different stages of childhood which potentially affects the educational outcomes in immediate life and the later life.
ii) Three binary measures (completed = 1, not completed = 0) of educational participation consisting of the following: completion of primary (standard V), middle (standard VIII), and secondary levels of education (standard X). Table 2 indicates the age group of respective samples for each of the outcomes mentioned.
iii) Performance in standard X at the secondary school leaving examination. These are reported for children, aged 15–40 years old, who have passed the examination, and results are reported within a range of three divisions (1^st^, 2^nd^, and 3^rd^, with 1^st^ being the best performance).
iv) STEM as the subject choice for the children in the age group of 15–40 years. This is a binary variable assuming a value of ‘1’ if the individual opted for a ‘STEM’ subject as the field of study at the higher secondary level (standard XI-XII), else it assumes a value of ‘0.’ We consider an individual’s field of study as STEM if she has enrolled in Science, Engineering, Vocational, or other Technical subjects. For robustness Table A3 in S1 Appendix, we also include commerce as a part of STEM, because a few subjects, such as statistics, involves knowledge of mathematical tools but are included in the commerce stream.

We use the district fixed effects ‘*δ*_*d*_’ throughout all our primary specifications to account for any time-invariant systematic differences across districts. To control for time-variant differences that may affect all the districts simultaneously, we include birth year fixed effects ‘*α*_*t*_’ while estimating the outcomes from the IHDS-2 data. However, while using the YLS data, controlling the time-varying factor is not possible. The children included in our analysis were born in 2001–2002, and all seven districts of YLS data were exposed to rainfall shocks in 2002. The potential concerns related to this have been discussed later in section 4.

We also control for the gender of the child in all our specifications. Among other covariates, ‘***H***_*h*_’ represents the vector of household level observables that could affect outcomes differently, which are: the father’s and mother’s education (if they have attended formal education = 1, and 0 otherwise) in the YLS sample, household head’s education (continuous variable measuring years of schooling completed) in the IHDS-2 sample, wealth status measured by the dummies of five quantiles (= 1 if household belongs to that quantile, and 0 otherwise) of the number of assets owned by the household (such as TV, fan, chair, etc.), number of members in the household, caste background (SC, ST, OBC or others), religion (Hindu, Muslim or others). Lastly, *ε*_*ithd*_ is *iid* the error term. Robust standard errors are used in the YLS data. We have not clustered the standard errors due to the small number of (seven) districts because inference based on standard errors produced by the clustering can sometimes be misleading if the number of clusters is small [43]. However, our results remain qualitatively the same even if we cluster the standard errors at the district level. To check for robustness, we also cluster the standard errors at the level of primary sampling units: Mandal. Our findings do not change due to clustering at the Mandal level (see Tables A15, A16 in S1 Appendix). In the IHDS-2 data, standard errors are corrected for heteroscedasticity by clustering them at the district levels.

**Fig 1:**
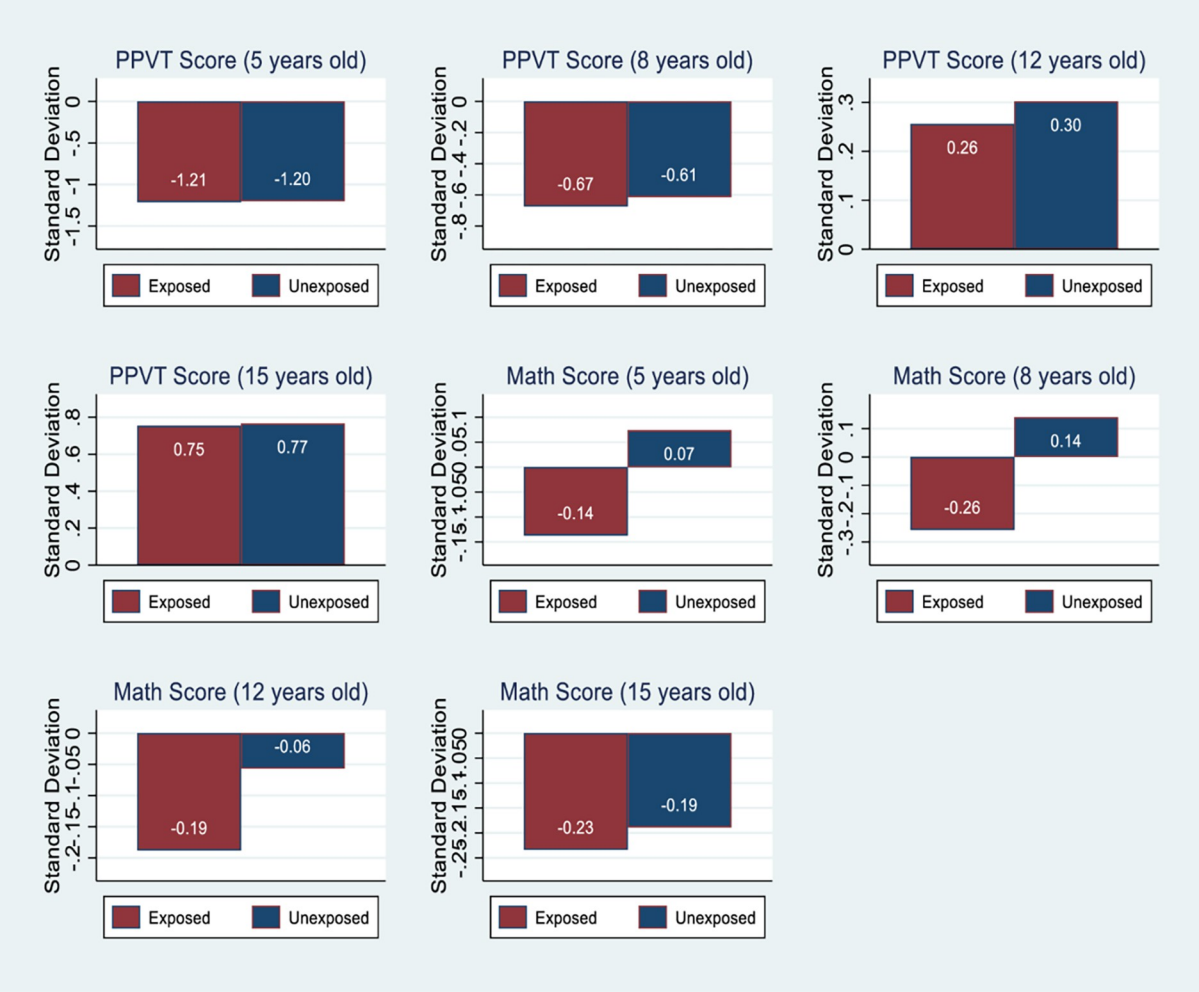
Test scores by the exposure to rainfall shock–YLS data.

**Fig 2:**
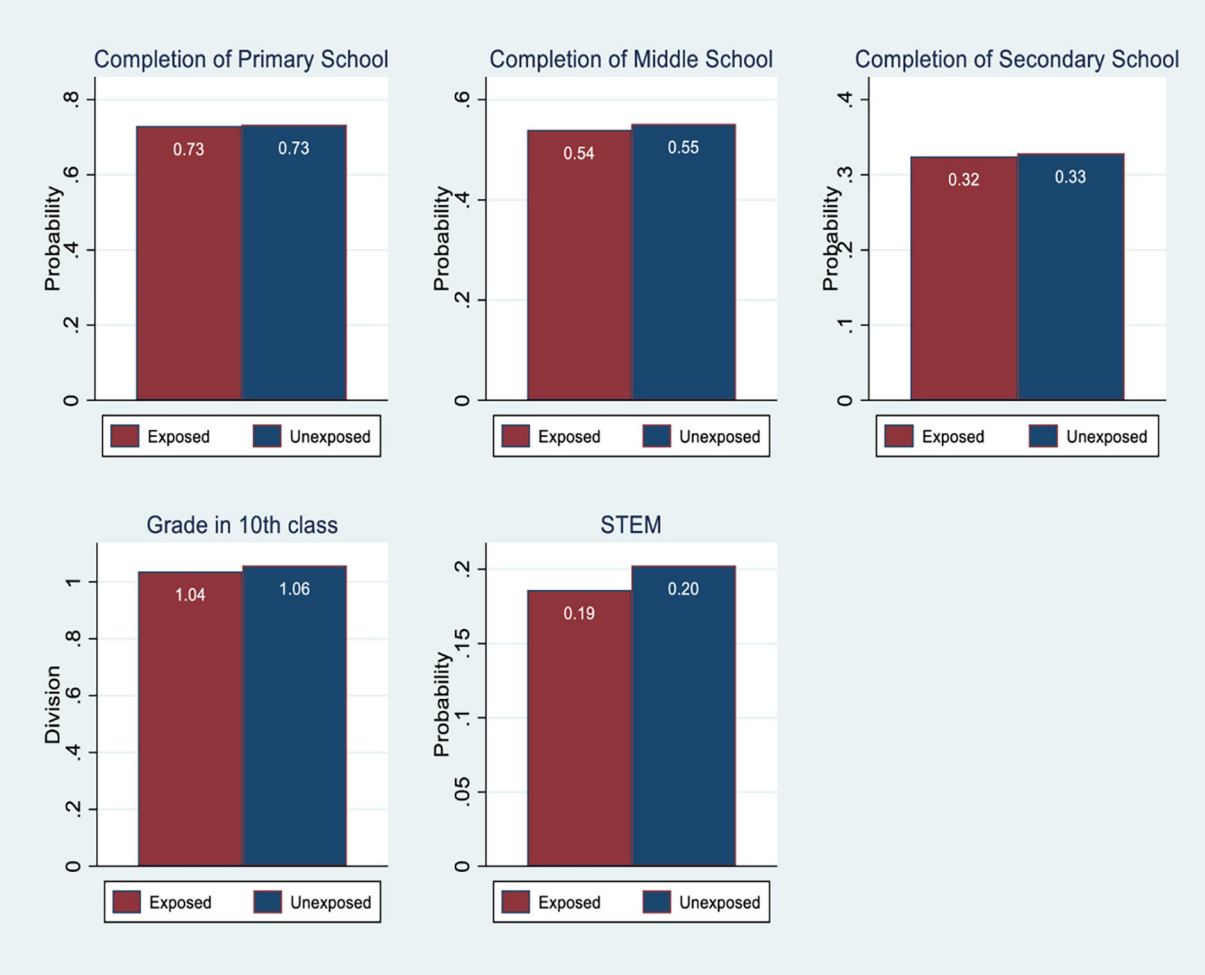
Educational outcomes by the exposure to rainfall shock–IHDS data.

Fig 1 shows the test scores of the children by their exposure to shock in the birth year. It shows that children exposed to shock in the birth year have lower test scores than those who are not exposed. Fig 2 shows that children exposed to negative rainfall shock have a lesser likelihood of completing middle school and secondary school, lower grades in 10^th^ standard, and a lower likelihood of opting for STEM in post-secondary education than their counterparts.

Tables 1 and 2 present descriptive statistics for variables and their difference in means between exposed and unexposed groups in YLS and IHDS-2 samples respectively. On average, in the YLS sample, the children exposed to the rainfall shocks have mothers with shorter heights (150.84 cm vs. 151.71 cm), belong to households with lesser members (5.38 vs. 5.74), more ST caste affilitaions (0.26 vs. 0.12) and followers of other religion (0.08 vs. 0.04), higher percentage of them have poorest (0.30 vs. 0.22) or poor wealth status (0.30 vs. 0.25), and more out of school children at the age of 5 years (0.90 vs. 0.96) and eight years (0.99 vs. 1.00). However, after controlling for all the above covariates and a within-district comparison in our fixed effects model, we expect to take care of the baseline differences between the affected and unaffected, if any.

**Table 1:** Difference in means of variables between samples exposed and unexposed to rainfall shock in the birth year: YLS Data.

Variables	Exposed	Unexposed	Overall	Difference	Standard Error of Difference
Male (= 1 if yes)	0.554	0.527	0.537	0.027	[0.029]
Father’s Formal Education	0.568	0.601	0.588	-0.033	[0.028]
Mother’s Formal Education	0.380	0.372	0.375	0.008	[0.028]
Mother’s Height (in cm)	150.840	151.710	151.369	-0.870**	[0.355]
Household Size	5.380	5.744	5.601	-0.364***	[0.139]
Caste					
SC (= 1 if yes)	0.204	0.207	0.206	-0.003	[0.023]
ST (= 1 if yes)	0.261	0.122	0.176	0.138***	[0.023]
BC (= 1 if yes)	0.406	0.518	0.474	-0.111***	[0.029]
OC (= 1 if yes)	0.129	0.153	0.144	-0.024	[0.020]
Religion					
Hindu (= 1 if yes)	0.899	0.930	0.918	-0.031*	[0.016]
Muslim (= 1 if yes)	0.024	0.029	0.027	-0.004	[0.009]
Others (= 1 if yes)	0.077	0.042	0.055	0.035**	[0.014]
Wealth Status					
Poorest (= 1 if yes)	0.297	0.218	0.249	0.079***	[0.025]
Poor (= 1 if yes)	0.303	0.247	0.269	0.056**	[0.026]
Middle (= 1 if yes)	0.196	0.286	0.251	-0.090***	[0.024]
Rich (= 1 if yes)	0.162	0.203	0.187	-0.041*	[0.022]
Richest (= 1 if yes)	0.042	0.046	0.044	-0.003	[0.012]
School Enrollment (= 1 if yes)					
Age 5	0.903	0.964	0.940	-0.061***	[0.015]
Age 8	0.986	0.999	0.994	-0.013**	[0.005]
Age 12	0.990	0.996	0.994	-0.006	[0.005]
Age 15	0.907	0.899	0.902	0.009	[0.017]
PPVT Scores					
Age 5	-1.211	-1.200	-1.204	-0.012	[0.047]
Age 8	-0.673	-0.614	-0.637	-0.059	[0.040]
Age 12	0.256	0.302	0.284	-0.046	[0.044]
Age 15	0.754	0.767	0.762	-0.013	[0.062]
Math Test Scores					
Age 5	-0.137	0.074	-0.008	-0.211**	[0.088]
Age 8	-0.257	0.140	-0.015	-0.397***	[0.068]
Age 12	-0.188	-0.057	-0.108	-0.131*	[0.078]
Age 15	-0.234	-0.189	-0.207	-0.044	[0.072]
Observations	495	769	1264	1264	

⁎⁎⁎ p < 0.01

⁎⁎ p < 0.05

⁎ p < 0.1

Note: Children who have faced shock in the birth year are considered exposed and those who have not faced are considered unexposed. *** p<0.01, ** p<0.05, * p<0.1

**Table 2:** Difference in means of variables between samples exposed and unexposed to rainfall shock in the birth year: IHDS Data.

Variables	Exposed	Unexposed	Overall	Difference	Standard Error of Difference
Male (= 1 if yes)	0.497	0.498	0.498	-0.001	[0.005]
Age (in years)	23.331	25.005	24.034	-1.674***	[0.091]
Household Head’s Education (in years)	4.213	4.409	4.296	-0.196***	[0.047]
Household Size	6.383	6.358	6.373	0.025	[0.032]
Caste					
SC (= 1 if yes)	0.185	0.186	0.186	-0.001	[0.004]
ST (= 1 if yes)	0.142	0.122	0.134	0.020***	[0.004]
BC (= 1 if yes)	0.420	0.413	0.417	0.007	[0.005]
OC (= 1 if yes)	0.253	0.279	0.264	-0.026***	[0.005]
Religion					
Hindu (= 1 if yes)	0.868	0.855	0.863	0.014***	[0.004]
Muslim (= 1 if yes)	0.077	0.088	0.082	-0.011***	[0.003]
Others (= 1 if yes)	0.055	0.057	0.056	-0.002	[0.002]
Wealth Status					
Poorest (= 1 if yes)	0.292	0.276	0.285	0.016***	[0.005]
Poor (= 1 if yes)	0.308	0.309	0.308	-0.001	[0.005]
Middle (= 1 if yes)	0.211	0.216	0.213	-0.005	[0.004]
Rich (= 1 if yes)	0.128	0.131	0.129	-0.002	[0.004]
Richest (= 1 if yes)	0.061	0.069	0.064	-0.008***	[0.003]
Completed Primary School (= 1 if yes): 11–40 years old	0.730	0.733	0.731	-0.003	[0.005]
Observations	20839	15087	35926	35926	
Completed Middle School (= 1 if yes): 14–40 years old	0.540	0.551	0.545	-0.012**	[0.006]
Observations	17481	13983	31464	31464	
Completed Secondary School (= 1 if yes): 16–40 years old	0.325	0.328	0.326	-0.004	[0.006]
Observations	15900	12578	28478	28478	
Grade in 10th class (= 0 if III, 1 if II, and 2 if I): 15–40 years old	1.037	1.058	1.046	-0.021	[0.013]
Observations	5028	4016	9044	9044	
STEM (= 1 if yes): 15–40 years old	0.186	0.203	0.193	-0.021	[0.013]
Observations	3785	3032	6817	6817	

⁎⁎⁎ p < 0.01

⁎⁎ p < 0.05

⁎ p < 0.1

Note: Children who have faced shock in the birth year are considered exposed and those who have not faced are considered unexposed.

On average, in the IHDS-2 sample, individuals who have faced rainfall shocks in the birth year are younger than their unaffected counterparts (23.33 years vs. 25.01 years). Comparing the household characteristics, we find that on average, children exposed to the shock belong to a family with lesser years of education completed by the household head (4.21 years vs. 4.41 years), with more having ST caste affiliations (0.14 vs. 0.12) and Hindu religious affiliations (0.87 vs. 0.86), and higher percentage of them with a poorest (0.29 vs. 0.28) wealth status.

One important point to note here is, that the information on exact birth year is missing for more than 75 percent of the individuals in the analytical sample (11–40 years old) of the IHDS-2 data. So, we use the year of the interview and the age of the individual to find the birth year of the individuals in the sample. Inaccuracy in reporting age may generate measurement error in the shock variable. We believe this is unlikely to be an issue in this study as we find a correlation of 0.98 between the constructed birth year variable and the available birth year variable.

## 3 Results

### 3.1 Impact on cognitive outcomes

The estimated coefficients of the rainfall shock on indicators of cognitive development are presented in Table 3, where columns are separated by different measures from the YLS data. The estimates in Panel A indicate that PPVT scores at ages 5 and 8 seem to be 0.18 and 0.07 standard deviation units lower for the children affected by the rainfall shock in their birth year compared to the unaffected cohort. The MAT scores at ages 5 and 8 seem to be 0.39 and 0.27 standard deviation units lower, respectively. We do not seem to find any significant impact on these measures for the 12 and 15-year-old children, and even with negative coefficients, the effect size seems very small.

**Table 3:** OLS estimates of rainfall shock in the birth year on test scores.

	PPVT				MAT			
	(1)	(2)	(3)	(4)	(5)	(6)	(7)	(8)
	Age 5	Age 8	Age 12	Age 15	Age 5	Age 8	Age 12	Age 15
***Panel A*: *Without School Enrollment Status***								
Shock in birth year	-0.180***	-0.074*	-0.049	-0.041	-0.385***	-0.269***	-0.072	-0.001
	[0.05]	[0.04]	[0.05]	[0.07]	[0.10]	[0.07]	[0.09]	[0.08]
Observations	1,264	1,264	1,264	1,264	1,264	1,264	1,264	1,264
R-squared	0.194	0.222	0.182	0.083	0.127	0.240	0.181	0.184
***Panel B*: *With School Enrollment Status***								
Shock in birth year	-0.182***	-0.060	-0.048	-0.058	-0.412***	-0.228***	-0.066	-0.036
	[0.05]	[0.04]	[0.05]	[0.07]	[0.10]	[0.07]	[0.08]	[0.07]
Observations	1,264	1,264	1,264	1,264	1,264	1,264	1,264	1,264
R-squared	0.194	0.231	0.184	0.118	0.130	0.267	0.186	0.280
Other Covariates	Yes	Yes	Yes	Yes	Yes	Yes	Yes	Yes
District FE	Yes	Yes	Yes	Yes	Yes	Yes	Yes	Yes

⁎⁎⁎ p < 0.01

⁎⁎ p < 0.05

⁎ p < 0.1

Notes: Robust standard errors in brackets. “Other covariates” include the gender of the child, father’s education, mother’s education, mother’s height, family size, religion, caste, and wealth status. In addition to these covariates, Panel B also includes an indicator variable equal to 1 for children enrolled in pre-school/school at the age when the test was conducted and 0 otherwise. ⁎⁎⁎ p < 0.01, ⁎⁎ p < 0.05, ⁎ p < 0.1.

Source: YLS Data

While the negative impact of an early life shock seems quite evident in childhood, it does not seem to persist in the later years. Hence, in the next stage, we investigate if there is any evidence of reinforcing investments by parents as one of the potential reasons for such fading out [15]. Since parental investment in schooling is expected to help cognitive development, we check the robustness of our estimates by including the current school enrollment status as a covariate. The estimates presented in panel B of Table 3 indicate lower PPVT scores for the affected children at age 5 by 0.18 standard deviation units. However, there seems to be some mitigation in cognitive development at age 8, where the effect size seems to be slightly smaller and insignificant after controlling for the school enrollment status. The impact on MAT scores at ages 5 and 8 seems to be lower for the affected cohort by 0.41 and 0.23 standard deviation units respectively. We do not seem to find any impact on the older children between 12 and 15 years of age. To summarize: One, as children grow older, we do not find any impact on their cognitive development even if they were affected by negative shock during their birth year. Two, a contemporaneous school enrollment, which could be a proxy for parental investment, could barely help alter the relationship between rainfall shocks in the birth year and cognitive development in later life.

What emerges from the above discussion is that the deficiencies acquired in early life seem to affect early life cognitive outcomes, which may not always be observed in later life outcomes. This mitigation may have happened due to the natural ability of children. To reiterate this mechanism of having an impact only in the early life and no effect in later life, as we control for the PPVT scores at age 5, we do not seem to find any impact of negative rainfall shock on PPVT scores at age 8, 12, and 15. As presented in panel A of Table 4 (the first three columns), the effects are negligibly small and statistically insignificant. As expected, we see strong positive associations of PPVT at five and PPVT at ages 8, 12 and 15. One standard deviation higher PPVT score at age five is associated with 0.25, 0.23, and 0.14 standard deviation units higher PPVT scores at age 8, 12, and 15, respectively. The last three columns of panel A (Table 4) support the same story. We do not seem to find any impact on MAT scores at age 12 and 15, even after controlling for the MAT score at age 5. However, the negative impact persists till age 8, with 0.19 standard deviation lower MAT scores for the affected children. Panel B of Table 4 shows that the estimates are robust to the current enrollment status in school. Overall, we find evidence that we may not see any impact in later life measures of cognitive development. After controlling for the cognitive abilities of the children in early life, the remaining differences in later life may completely disappear.

**Table 4:** OLS estimates of rainfall shock on test scores after controlling for test scores at the age of 5 years.

	PPVT			MAT		
	(1)	(2)	(3)	(4)	(5)	(6)
	Age 8	Age 12	Age 15	Age 8	Age 12	Age 15
***Panel A*: *Without School Enrollment Status***						
Shock in birth year	-0.029	-0.008	-0.015	-0.191***	-0.003	0.062
	[0.04]	[0.05]	[0.07]	[0.07]	[0.08]	[0.08]
PPVT score (Age 5)	0.251***	0.230***	0.143***			
	[0.03]	[0.03]	[0.04]			
MAT score (Age 5)				0.203***	0.179***	0.165***
				[0.02]	[0.02]	[0.02]
Observations	1,264	1,264	1,264	1,264	1,264	1,264
R-squared	0.287	0.233	0.092	0.299	0.217	0.219
***Panel B*: *With School Enrollment Status***						
Shock in birth year	-0.016	-0.006	-0.034	-0.150**	0.002	0.023
	[0.04]	[0.05]	[0.07]	[0.06]	[0.08]	[0.07]
PPVT score (Age 5)	0.249***	0.230***	0.133***			
	[0.03]	[0.03]	[0.04]			
Math score (Age 5)				0.203***	0.177***	0.149***
				[0.02]	[0.02]	[0.02]
Enrolled	0.809	0.371***	0.681***	2.478***	1.160*	1.322***
	[0.53]	[0.12]	[0.11]	[0.75]	[0.63]	[0.16]
Observations	1,264	1,264	1,264	1,264	1,264	1,264
R-squared	0.296	0.234	0.126	0.326	0.222	0.307
Other Covariates	Yes	Yes	Yes	Yes	Yes	Yes
District FE	Yes	Yes	Yes	Yes	Yes	Yes

⁎⁎⁎ p < 0.01

⁎⁎ p < 0.05

⁎ p < 0.1

Notes: Robust standard errors in brackets. “Other covariates” include the gender of the child, father’s education, mother’s education, mother’s height, family size, religion, caste, and wealth status. In addition to these covariates, Panel B also includes an indicator variable equal to 1 for children enrolled in pre-school/school at the age when the test was conducted and 0 otherwise. ⁎⁎⁎ p < 0.01, ⁎⁎ p < 0.05, ⁎ p < 0.1. Source: YLS data

### 3.2 Impact on education outcomes

To follow up on this finding, we estimate the impacts on schooling outcomes at different stages of children’s lives using the IHDS-2 data. Suppose cognitive ability in early life is the deciding factor in later life performances. In that case, we do not expect the schooling outcomes to be affected in later life as children with lower cognitive abilities are expected to drop out. The estimate from the following specification is presented in Table 5.

**Table 5:** OLS estimates of rainfall shock in the birth year on educational outcomes.

	(1)	(2)	(3)	(4)	(5)
	Completed Primary School	Completed Middle School	Completed Secondary School	Grade	STEM
Shock in birth year	-0.013*	-0.017*	-0.020**	-0.030	-0.001
	[0.01]	[0.01]	[0.01]	[0.02]	[0.01]
Observations	35,926	31,464	28,478	9,044	6,817
R-squared	0.297	0.334	0.291	0.232	0.289
Other Covariates	Yes	Yes	Yes	Yes	Yes
District FE	Yes	Yes	Yes	Yes	Yes
Age FE	Yes	Yes	Yes	Yes	Yes

⁎⁎⁎ p < 0.01

⁎⁎ p < 0.05

⁎ p < 0.1

Notes: Robust standard errors in brackets are clustered at the district level. “Other covariates” include gender, household head’s education, family size, religion, caste, and wealth status.

Source: IHDS data


$$Y_{ithd}=\beta_{0}+\beta_{1}({Shockinbirthyear}_{dt})+\alpha_{t}+{\gamma Male}_{i}+{\theta H}_{h}+\delta_{d}+\varepsilon_{ithd}$$ (2)

Where, the dependent variable, *Y*_*ithd*_ represents alternative measures of education outcomes from the IHDS-2 data as presented earlier. All other variable specifications are the same as presented in the data section earlier.

The estimates of different education outcomes are separated by columns in Table 5, where the first three columns present the impact on the completion of primary, middle, and secondary education respectively. We find that for the affected children, the likelihood of completion of primary, middle and secondary education are lesser by 1, 2 and 2 percentage points respectively, as compared to their unaffected counterparts.

An interesting fact emerges thereafter as we investigate subject choices after secondary school using the IHDS-2 data. We do not seem to find any differential impact for the affected cohort (presented in column 4 of Table 5) on high school grades. As discussed earlier, the children with first division in the 10^th^ class seem to be 20 percentage points more likely to choose STEM subjects in high secondary school. Hence, this also leads to the fact that in column 5, we do not see any impact for the affected cohort on the likelihood of choosing STEM at the higher secondary level. It is important to note here that the high school grades considered in column 4 are observed only for the students who are able to complete secondary education. Since the likelihood of completion of secondary education is lower for the affected cohort, we do not observe the high school grades for a significant number of affected individuals. Therefore, it is expected that we may not observe any impact on the selected sample and the same logic spills over to the subject choice outcome, which is decided by the completion of secondary school.

However, when we explore the later life education outcomes using the IHDS data, the potential selection bias needs further attention. Individuals in the potential treatment group (exposed to rainfall shocks) are more likely to drop out of the education system as compared to the control group (unexposed to rainfall shocks). We try to address this selection bias by estimating the bounds of the treatment effects accounting for this later selection. We have estimated the Lee [31] bounds on the effect of negative rainfall shock on the two outcome variables, ‘Grade’ and ‘STEM’, that are likely to suffer from the later selection. The objective of the Lee [31] bounds procedure is to attain the same level of attrition rate in both the treatment and control groups by trimming the outcome distribution of the group with a lesser proportion of attrition.

These bounds are estimated under two assumptions: a) exogeneity of the negative rainfall shocks and b) monotonicity of the selection mechanism. Exogeneity of the negative rainfall shocks requires that the exposure to the negative rainfall shocks be independent of their potential outcomes. We believe this assumption is justified in our context as we measure these rainfall shocks in the birth year at the district level. Exposure to these shocks is likely to be independent of the potential outcomes measured after 15 years of birth i.e., grade received in 10^th^ standard and choice of STEM in post-secondary school. Monotonicity of the selection mechanism implies that exposure to the treatment can affect the selection mechanism in “one direction” [31]. Specifically, it excludes the possibility that exposure to negative rainfall shocks during the birth year increases the likelihood of some individuals dropping out of school and simultaneously increase the likelihood of others to join school. It is unlikely that exposure to negative rainfall shocks at birth can increase the likelihood of joining school. Hence, we believe the assumption of monotonicity is plausible in this context.

Under these two assumptions, Lee [31] bounds are estimated by trimming the outcome distribution of the group (treatment or control) with the lesser proportion of attrition [44] due to later selection. Individuals aged 15–40 years old and dropped out from school before the completion of 10^th^ (11^th^) standard, are considered as attrition in the sample analysing the grade received in 10^th^ standard (STEM in post-secondary school). The base used to calculate the proportion of attrition is equal to the sum of these observations of attrition for each of the outcome variables and the number of individuals aged 15–40 years old and completed 10^th^ (11^th^) standards or more with no missing values of the outcome variable (which is, grade received in 10^th^ standards or STEM in post-secondary school). The proportion of attrition in the treatment group (P_T = 1_) is 57.79 (68.49) percent and in the control group (P_T = 0_) is 56.82 (67.57) percent for grade received in 10^th^ standards (STEM in post-secondary school). Since the proportion of attrition is lower in the control group relative to the treatment group, we trim the outcome distribution of the control group by proportion P = $\frac{P_{T=1}-P_{T=0}}{\left(1-P_{T=0}\right)}$ from the lower (upper) end such that [31] lower (upper) bound of the treatment effect is estimated, which is presented Table A4 in S1 Appendix. The Lee bounds are implemented in Stata 16 using the *leebounds* command [45].

The overall effect estimated from our naïve specification captures the effect of negative rainfall shock during birth when bias due to selection in later stages is not taken into consideration. Lee bounds results based on the naïve specification show that the lower bound of the effect of negative rainfall shocks on grade and STEM is significantly negative, with the upper bound remaining inconclusive. Since the lower bound, after taking care of the potential sample selection or attrition bias seems significantly negative, it reiterates the argument that the ones who drop out early might be the ones with potentially poor outcomes. Without being able to see them in our sample when we measure the long-run impact, the impact does not seem to be significantly negative. It is important to mention here that for performing Lee bounds analysis along with conditioning on other covariates, one would require to trim samples within each category of the covariates. Due to insufficient variations within the discrete categories, we could not control for other covariates while estimating the Lee bounds.

### 3.3 Heterogeneous effects across gender

Table 6 presents the differential impacts of shock by gender. The estimates indicate that the average MAT score of affected males at age 5 is 0.27 standard deviation unit less than affected females, and that seems to be the only differential impact among all measures of cognitive abilities. The only other differential effects found are in the completion of primary, middle and secondary education, where the likelihood of completing the respective standards are 0.5, 0.4, and 0.3 points lesser (respectively) for the males as compared to the females. Although, an early life rainfall shock is found to cause higher mortality among women [46], the surviving girls in India seem to perform better than the boys in school attainments, without much differential effects on cognitive outcomes.

**Table 6:** OLS estimates of rainfall shock and its interaction with the gender on test scores and educational outcomes.

	(1)	(2)	(3)	(4)	(5)	(6)	(7)	(8)
***Panel A*: *YLS***	PPVT				MAT			
	Age 5	Age 8	Age 12	Age 15	Age 5	Age 8	Age 12	Age 15
Shock in birth year*Male	-0.059	-0.049	-0.043	-0.095	-0.269*	-0.094	-0.139	-0.211
	[0.09]	[0.07]	[0.08]	[0.13]	[0.16]	[0.12]	[0.14]	[0.13]
Shock in birth year	-0.148**	-0.047	-0.026	0.012	-0.237*	-0.218**	0.005	0.114
	[0.07]	[0.06]	[0.06]	[0.11]	[0.13]	[0.09]	[0.11]	[0.11]
Male	0.078	0.171***	0.157***	0.164**	0.182*	0.122	0.202**	0.368***
	[0.05]	[0.05]	[0.05]	[0.07]	[0.11]	[0.07]	[0.09]	[0.09]
Observations	1,264	1,264	1,264	1,264	1,264	1,264	1,264	1,264
R-squared	0.195	0.222	0.183	0.083	0.129	0.240	0.181	0.186
Other Covariates	Yes	Yes	Yes	Yes	Yes	Yes	Yes	Yes
***Panel B*: *IHDS- Level Completion***	Primary	Middle	Secondary	Grade	STEM			
Shock in birth year*Male	-0.046***	-0.041**	-0.031*	-0.008	0.026			
	[0.01]	[0.02]	[0.02]	[0.03]	[0.02]			
Shock in birth year	0.010	0.003	-0.005	-0.025	-0.017			
	[0.01]	[0.01]	[0.01]	[0.03]	[0.02]			
Male	0.169***	0.192***	0.160***	-0.018	0.146***			
	[0.01]	[0.01]	[0.01]	[0.02]	[0.02]			
Observations	35,926	31,464	28,478	9,044	6,817			
R-squared	0.298	0.334	0.291	0.232	0.289			
Other Covariates	Yes	Yes	Yes	Yes	Yes			
Age FE	Yes	Yes	Yes	Yes	Yes			

⁎⁎⁎ p < 0.01

⁎⁎ p < 0.05

⁎ p < 0.1.

Notes: In Panel A, robust standard errors are reported in brackets, and “Other covariates” include the father’s education, mother’s education, mother’s height, family size, religion, caste, and wealth status. In Panel B, robust standard errors in brackets are clustered at the district level, and “Other covariates” include the household head’s education, family size, religion, caste, and wealth status. In both panels, the male is a binary variable indicating whether the child belongs to the male cohort (= 1) or the female cohort (= 0). District fixed effects are included in both panels.

### 3.4 Falsification

In order to ensure that our results are not mere estimations of any general trend across time or space, we conduct the following falsification test. We estimate the impact of rainfall shock that occurred five years before the birth of the child instead of rainfall shock in the birth year. Using this new exposure variable, we re-estimate specifications (1) and (2) and present estimates for both YLS and IHDS-2 samples in panels A and B of Table 7 respectively. As expected, none of the coefficients in either of the panels seems to be significant when we use our primary specification of rainfall shock.

**Table 7:** Falsification Test: OLS estimates of rainfall shock in false birth year.

	(1)	(2)	(3)	(4)	(5)	(6)	(7)	(8)
***Panel A*: *YLS***	PPVT				MAT			
	Age 5	Age 8	Age 12	Age 15	Age 5	Age 8	Age 12	Age 15
Shock in five years before birth	-0.053	-0.027	0.007	0.016	0.037	0.001	-0.052	-0.022
	[0.06]	[0.05]	[0.05]	[0.07]	[0.12]	[0.09]	[0.10]	[0.09]
Observations	1,264	1,264	1,264	1,264	1,264	1,264	1,264	1,264
R-squared	0.186	0.220	0.182	0.083	0.116	0.231	0.180	0.184
Other Covariates	Yes	Yes	Yes	Yes	Yes	Yes	Yes	Yes
District FE	Yes	Yes	Yes	Yes	Yes	Yes	Yes	Yes
***Panel B*: *IHDS***	Completed Primary School	Completed Middle School	Completed Secondary School	Grade	STEM			
Shock in five years before birth	-0.005	-0.003	-0.013	0.028	0.010			
	[0.01]	[0.01]	[0.01]	[0.02]	[0.02]			
Observations	35,926	31,464	28,478	9,044	6,817			
R-squared	0.297	0.334	0.290	0.232	0.289			
Other Covariates	Yes	Yes	Yes	Yes	Yes			
District FE	Yes	Yes	Yes	Yes	Yes			
Age FE	Yes	Yes	Yes	Yes	Yes			

⁎⁎⁎ p < 0.01

⁎⁎ p < 0.05

⁎ p < 0.1.

Notes: In Panel A, robust standard errors are reported in brackets, and “Other covariates” include the gender of the child, father’s education, mother’s education, mother’s height, family size, religion, caste, and wealth status. In Panel B, robust standard errors in brackets are clustered at the district level, and “Other covariates” include gender, household head’s education, family size, religion, caste, and wealth status.

## 4 Potential concerns

In the next few paragraphs, we discuss a few potential concerns regarding our measure of the treatment variable and our way of addressing those.

First, so far, we have considered the annual average rainfall in the first year after birth. One could also measure the shock by in utero-trimester level and extend the rainfall shock to the next two years after birth. However, we do not find sufficient variation in the exposure to rainfall shocks during different trimesters due to a limited number of (seven) districts in the survey. Specifically, 95%, 96%, and 94% of the children in the analytical sample are exposed to negative rainfall shocks during the first, second, and third trimesters of the gestation period. Due to this, we are unable to derive any conclusive evidence while measuring the shock during gestation.

However, following the literature on the importance of the first 1000 days of life, as robustness checks, we have included two additional shock variables measured one year after birth and two years after birth. Estimates presented Tables A9-A11 in S1 Appendix indicate that the effects of rainfall shock during the birth year remain qualitatively unchanged even after controlling for the above two shocks. We do not seem to find any significant impact of negative rainfall shock at ages one and two on PPVT and MAT test scores measured at the different ages, except for PPVT at age eight (-0.17), MAT at age 12 (-0.38), and 15 (-0.63 due to shock at age 1, -0.56 due to shock at age 2). However, the effects of negative rainfall shocks in the birth year on MAT at age 15, shocks in the second year on PPVT at age eight, and on MAT at age 15, also turn inconclusive after the inclusion of the current school enrolment status as a covariate. Moreover, we do not find a significant effect of negative rainfall shock at ages one and two on educational outcomes except in two cases, that are, the rainfall shock at age one on completion of secondary school (-0.02) and the rainfall shock at age two on grade (-0.05).

Second, another related concern in measuring the treatment variable would be that the rainfall shock measure is defined as the difference between the logarithm of current year rainfall and long-term average rainfall. This is a deviation from the standard literature, which often uses a threshold cut-off to ensure that minor deviations from the long-term average do not count as shocks. Maccini and Yang [21] have used a continuous measure of deviations in rainfall. Shah and Steinberg [16] have used the major deviations to construct the shock variable such that it takes a value of +1 if the yearly rainfall is above the 80^th^ percentile, -1 if the yearly rainfall is below the 20^th^ percentile and 0 otherwise. We have followed Maccini and Yang [21] to construct the continuous measure of deviations in rainfall. We used this continuous measure to construct our binary measure of negative rainfall shock. However, we also check the robustness of our estimates using extreme negative rainfall shocks instead. Following Shah and Steinberg [16], we reconstruct the rainfall shock that takes a value of one if the yearly rainfall is below the 20^th^ percentile and 0 otherwise. Estimates based on this newly defined shock variable are presented Tables A12-A14 in S1 Appendix.

The new estimates using the YLS data remain qualitatively unchanged. Instead, the magnitudes of the coefficients have increased. However, the effects of rainfall shocks on other educational outcomes are no longer conclusive. We believe this might have happened because the new control group consists of individuals who might have been exposed to some amount of rainfall shocks, attenuating the difference with the treatment group. For example, individuals born in districts where the rainfall in the birth year may have been in the range of 20^th^ and 30^th^ percentile are part of our new control group now. Hence, the overall effects of negative rainfall shocks on educational outcomes turn inconclusive.

Using this alternative measure of extreme shock too, we do not seem to find any evidence of differential impact across gender in terms of the cognitive outcomes (Table A17 in S1 Appendix). The differential effects on completion of primary and secondary schools being very similar across alternative measures of rainfall shocks, we do not seem to find any differential impact on the completion of middle schools. As explained earlier, the potential reasons for these minor differences with respect to alternate shock measures could be two-fold: first, some of the observations from the control groups in the extreme shock measure may have been part of the treated group in our primary specification because of the way of its construction. Second, even then, the estimates of YLS data seem robust. The only few variations noticed in IHDS data could be because of estimating longer-run outcomes, where the potential bias discussed earlier are much higher.

We also present the falsification estimates for the alternatively used extreme rainfall shock measures Table A18 in S1 Appendix. As expected, none of the coefficients in either of the panels are negative as well as statistically significant. The Lee bound estimates using these alternative measures are presented Table A19 in S1 Appendix, where we find statistically significant negative bounds similar to our primary specification.

Third, one may be concerned about the limited variability of rainfall data, which is measured at the district level. Since YLS does not provide geo codes, and the community level identifiers (that would be below the district level) are not made public to the researchers, we are unable to merge the external rainfall data (that is originally available at the grid level) below the district level. We provide a few statistics on the correspondence between distance in Km. and the grid levels in appendix section B1. However, this generates a possibility of unknown measurement error in our estimates because of the comparatively large size of Indian districts.

Fourth, related to the above-mentioned lack of variation in the rainfall data at the district level, we are unable to control for any time-varying factors. Our work using IHDS data has age-fixed effects. The YLS estimates do not have age-fixed effects because in the year 2002, all districts got exposed, and in the year 2001 only two districts got exposed. Since our treatment variable varies at the district-year level, the quarter-of-age dummy does not have enough variation. This is because all four quarters of 2002-born in all seven districts are treated, and all four quarters of 2001-born from five (out of seven) districts are the control group. Then, the only way a 3-6-month-old cohort could be compared was if this cohort was not affected by rainfall shock in the year 2001 (since all were affected in the year 2002), and that means she was born in one of the five control districts of 2001. A cross-tabulation (available with authors on request) reveals barely any variation across cohorts in this dimension. Hence, our specification could not control for that. Chang et. al. [36] use community-by-month fixed effects because they have got access to the community identifiers, facilitating them to merge rainfall data with the community level YLS data, with variation across communities in rainfall in both years.

However, we believe that the absence of time-varying control may not be able to drive our results completely because of the following reasons: first, it would be a concern if there had been a major change in the district-level infrastructures between the two years that could affect the children’s exposure to rainfall differently. However, this is unlikely to be a concern as the children in our analysis are born between April 2001 and May 2000. The likelihood of a major change in the district-level infrastructure within a year seems negligible. It is important to mention here that Dasgupta, [47] has also not controlled for birth year fixed effects while evaluating the role of the National Rural Employment Guarantee Scheme (NREGS) in mitigating the negative effects of early life rainfall shocks on long-term health outcomes using YLS data. Second, if we had used raw-test scores, chances would be higher that we were comparing an older child having the natural ability of better performance with a younger child who was yet to catch up. Age-standardized test scores are expected to take out the variability in natural ability due to age. Third, since the primary findings of our IHDS estimates, in the long run, conform to the primary findings of the YLS estimates, in the long run, we do not expect the negative estimates of YLS data to be driven by the lower age-standardized test scores of 2002-born children.

Other than potential concerns regarding the construction of treatment variables as mentioned above, one may worry about the potential bias due to sample attrition. The first type of attrition may happen due to mortality, which would primarily contribute to the problem of morality selection-related bias. We are unable to quantify the magnitude of the bias arising from mortality selection. Information on retrospective historical fertility of women would provide us some idea on infant mortality due to rainfall shocks, but none of the data provides such a module. Kumar et al. [48] use Indian data to examine the effects of a drought year, defined as the year in which monsoon rainfall is below 75 percent of the historical average in a particular district, on infant mortality rate. They find that being exposed to negative rainfall shocks increases the infant mortality rate by 3.5 per 1000 live births. However, this kind of selection can potentially affect our analysis of IHDS data only and is unlikely to be a concern in the analysis based on the YLS data. Because, for the latter, the tests are conducted for all the surveyed children irrespective of their school participation status.

The second type of problem contributing to attrition bias may arise due to the 12.8% sample attrition in the YLS data. We attempt to present an idea about the potential bias through the following mechanisms.

First, we assess the possibility of selective attrition by using the data from the first round (2002) to compare the individual and household level characteristics of children in the analytical sample and children excluded. The exclusion happened either due to unavailability in remaining rounds, or due to missing data in outcome variables and other covariates (Table A5 in S1 Appendix). On average (see Table A5 in S1 Appendix), children in the analytical sample have fathers (0.59 vs. 0.42) and mothers (0.38 vs. 0.20) with a higher likelihood of formal education, belong to Muslim religion (0.03 vs. 0.01) and a lesser proportion of them belong to poorest wealth status (0.25 vs. 0.33). There is no significant difference between the two samples across the remaining characteristics. This analysis suggests weak evidence of systematic attrition. However, it cannot be ruled out completely.

Second, we try to correct for potential attrition bias by utilizing the inverse probability weighting (IPW) technique following Mondi et al. [49]. It helps us to evaluate the most efficient coefficient estimates after accounting for potential attrition bias [50, 51]. We begin with estimating a logistic regression of a binary variable, indicating whether a particular child is included in the analytical sample (= 1) or not (= 0), on a set of predictors that are likely to determine the likelihood of attrition. We include the gender of the child (= 1 if male, 0 otherwise), any antenatal visit during pregnancy (= 0 if no, 1 if yes, 2 if not known or information not available), and whether the child met with serious injury or illness (= 1 if yes, 0 otherwise), number of children born to the mother, father’s and mother’s education (, assumes a value of 0 if never attended school, 1 if attended, 2 if not known or information not available), wealth status measured by the dummies of five quantiles (= 1 if household belongs to that quantile, and 0 otherwise) of the number of assets owned by the household, number of members in the household, dummy indicating the missing data on wealth status (= 1 if wealth is not available and 0 otherwise), caste, religion and district dummies. These individual and household level covariates reflect the socioeconomic status of the children and are likely to determine the likelihood of attrition. The marginal effects based on this logistic model are presented Table A6 in S1 Appendix.

Among the predictors, male children have a higher likelihood of being in the analytical sample as are those children whose mothers and fathers have complete formal education and belong to the Muslim religion. Missing information on antenatal visits during pregnancy and residence in the YSR district are negatively associated with the likelihood of inclusion in the analytical sample.

Using these results, we estimate the predicted probability (*p*) of being in the analytical sample. Inverse probability weights are calculated based on the inverse of this predicted probability. Specifically, these weights are equal to *1/p* if a particular observation is included in the analytical sample and *1/(1-p)* if a particular observation is not included in the analytical sample. These inverse probability weights are used as the probability weights in the least squares regression analysis of rainfall shocks on test scores. We examine the robustness of our results in Tables 3 and 4 after using these inverse probability weights as probability weights. Our results are robust to the inclusion of inverse probability weights (presented Tables A7 and A8 in S1 Appendix respectively, replicating the same specifications of Tables 3 and 4).

## 5 Concluding discussion

In our attempt to explain the mixed findings of early life negative shock on long-run outcomes, we estimate the impact of a negative rainfall shock on a series of outcomes connected to the educational pursuits of children. Beginning from the cognitive development at age 5, we estimate the impact of a negative rainfall shock on cognitive development till adolescence, followed by education participation till high school, and the subject choice. We use the YLS data from the state of India and the nationally representative IHDS-2 data to capture outcomes at different stages.

While the negative impacts of the shock on the cognitive development of children at ages 5 and 8 are quite strong, we are unable to find evidence of impacts on the cognitive development of children at the later stages. The seemingly insignificant effects continue to secondary-school grade or in subject choice beyond secondary school, whereas the likelihood of educational participation at different levels is lower for the affected group. The most interesting among these findings is, that the moment we control for cognitive development at age 5, the impact at age 8 seems to attenuate. This indicates that the negative impact on early life has a dampening effect on cognitive development in later life. A similar negative effect persists in education attainments till secondary school.

However, as we do not observe all the affected children, when we analyze the long-run education outcomes on secondary grades or subject choice, we do not seem to find any impact. Indian students need to compete through high stake national-level exams at the end of their higher secondary school, for their admissions into engineering, medical schools, or prestigious colleges for natural sciences, and studying STEM at the higher secondary level is a mandatory requirement for that. Therefore, understanding the nuances of analyzing the impacts of early life shocks on the likelihood of opting for STEMS becomes crucial too. But as we attempt to take care of the potential sample selection bias or attrition bias, the Lee bounds estimates present a significant negative impact at the lower bound for both measures of the rainfall shocks (with Table A19 in S1 Appendix having the bounds for the extreme shock specification). This provides the supporting evidence that the ones who drop out from the education system, seems to be the ones driving the null impact in the long run.

Our findings conform to some of the existing studies where the impact of the negative shock on long-run education outcomes are indeed insignificant [16]. Our findings can also be added to the literature which establishes that the timing of shock may matter a great deal [9, 12, 52].

However, the findings should be interpreted with caution for a few reasons. We do not observe all individuals when we estimate the impact on their grades in secondary schools. The children we observe are the ones who have passed the secondary examination successfully, and who may be better off than others with respect to cognitive ability. So, the true estimate would potentially indicate a more negative impact than what we end up observing. Similar issues arise for subject choice outcomes too.

Also, while explaining this seemingly puzzling finding one should note that in developing countries like India, school attainment is not a good measure of the quality of education. With minimal or no cost for attending public-funded primary and middle schools, the attainment is expected to be much higher as compared to a situation where one could capture the variations in quality [53]. Hence, the weak association between cognitive ability in later years and schooling outcomes is quite possible.

Overall, although this work opens the nuances of measuring the impact of early life shocks on later life education outcomes, it is able to re-establish the existing finding that cognitive development in early childhood is indeed a crucial channel toward long-run educational pursuits. It is important to note here that there are myriad ways through which individuals, households, or communities invest in strategies designed to mitigate the impacts of such shocks [54]. Apart from resilience, there are several aspects of behavioral dimensions that make a long-run outcome quite diverse in nature, which makes the generalizability of an impact difficult in the long run.

## Supporting information

S1 Appendix (DOCX)
