# Supplementary material for: Impact of early life shocks on educational pursuits–Does a fade out co-exist with persistence?
Source: PLoS One. 2022 Oct 13;17(10):e0275871. doi: 10.1371/journal.pone.0275871 (PMC9681159; doi:10.1371/journal.pone.0275871)
Supplement: S1 Appendix — (DOCX) [file pone.0275871.s001.docx]

**Impact of Early Life Shock on Educational Pursuits – Does a Fade out Co-exist with Persistence?**

**Appendix Section A: Tables and Figures**

Figure A1. Schematic representation of the children included in the study.


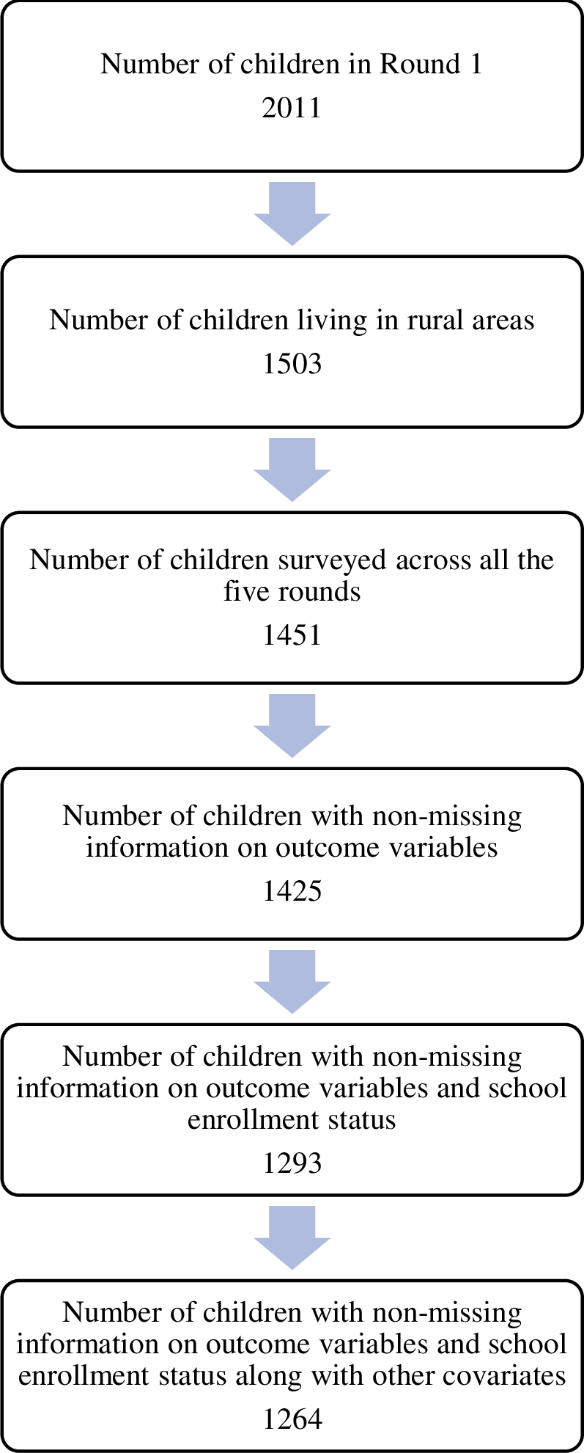


Notes: Although, the YLS surveys 2011 children in the first wave, our targeted sample is restricted to 1451 children surveyed across all the rounds and born in the rural region (see figure A1). However, our analytical sample reduces to 1264 due to missing information on outcome variables, school enrolment status and other covariates. Using 1451 as the base, we report (1-(1264/1451)) = 12.8% as the attrition due to missing information. Even if we change our base from 1451 to 1503 (with the latter being the number of children born in rural region) for calculating the attrition rate, the reported figure would be 15.9% [=1-(1264/1503)].

Figure A2. Rainfall Distribution in the year of birth.


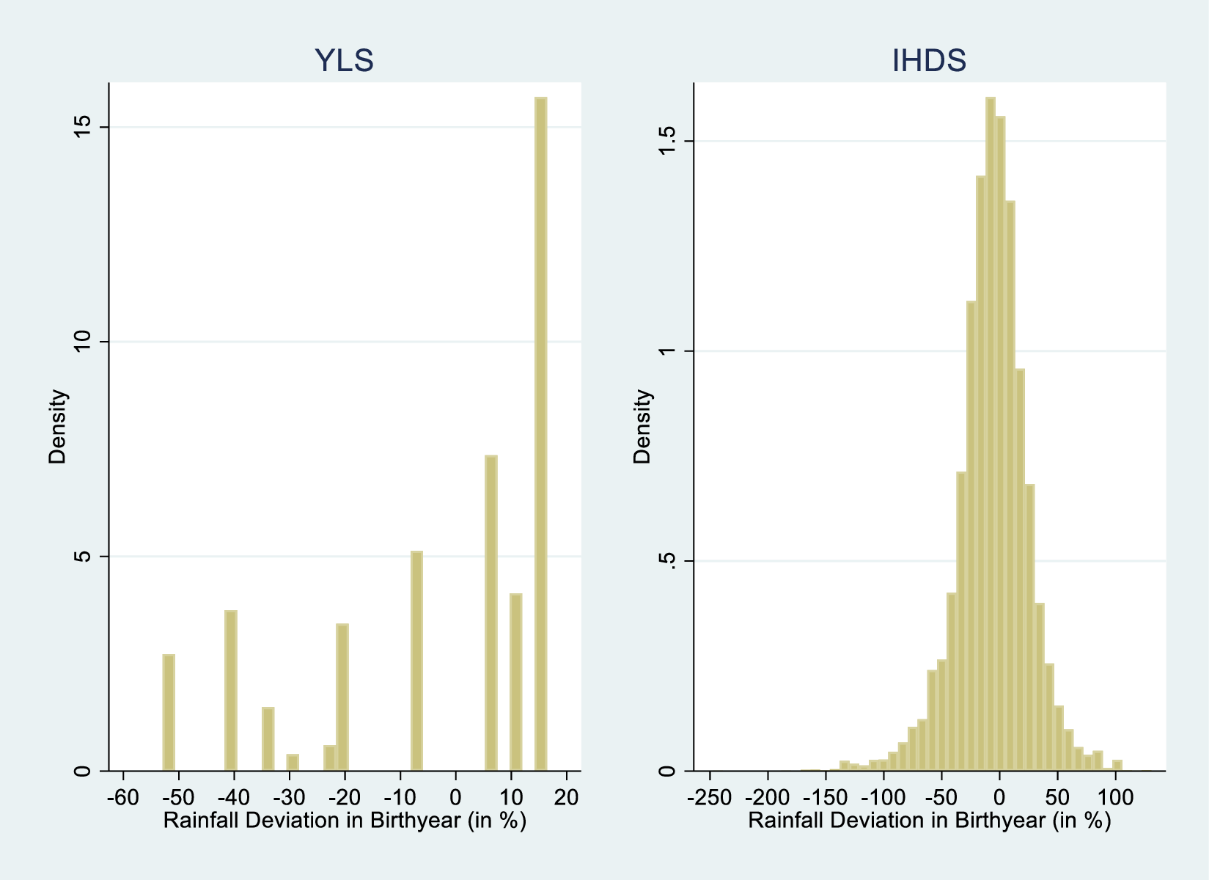


Table A1. OLS estimates of rainfall shock on test scores - Results for all the covariates along with the main variable of interest in Panel A of table 2.

|  | (1) | (2) | (3) | (4) | (5) | (6) | (7) | (8) |
| --- | --- | --- | --- | --- | --- | --- | --- | --- |
|  | PPVT | | | | MAT | | | |
|  | Age 5 | Age 8 | Age 12 | Age 15 | Age 5 | Age 8 | Age 12 | Age 15 |
| Shock in birth year | -0.180*** | -0.074* | -0.049 | -0.041 | -0.385*** | -0.269*** | -0.072 | -0.001 |
|  | [0.05] | [0.04] | [0.05] | [0.07] | [0.10] | [0.07] | [0.09] | [0.08] |
| Male | 0.055 | 0.152*** | 0.141*** | 0.127** | 0.077 | 0.085 | 0.147** | 0.286*** |
|  | [0.04] | [0.04] | [0.04] | [0.06] | [0.08] | [0.06] | [0.07] | [0.07] |
| Father's Formal Education | 0.187*** | 0.056 | 0.132*** | 0.180*** | 0.292*** | 0.146** | 0.257*** | 0.242*** |
|  | [0.04] | [0.04] | [0.04] | [0.07] | [0.09] | [0.07] | [0.08] | [0.07] |
| Mother's Formal Education | 0.270*** | 0.250*** | 0.211*** | 0.094 | 0.396*** | 0.429*** | 0.396*** | 0.450*** |
|  | [0.05] | [0.04] | [0.04] | [0.07] | [0.09] | [0.06] | [0.08] | [0.07] |
| Mother's Height | 0.002 | 0.001 | 0.002 | -0.003 | -0.002 | 0.010** | 0.008 | 0.010** |
|  | [0.00] | [0.00] | [0.00] | [0.01] | [0.01] | [0.00] | [0.01] | [0.01] |
| Household Size | -0.003 | -0.001 | 0.002 | 0.005 | 0.009 | -0.012 | -0.012 | -0.001 |
|  | [0.01] | [0.01] | [0.01] | [0.01] | [0.02] | [0.01] | [0.02] | [0.01] |
| Caste: SC | -0.145** | -0.125* | -0.112 | -0.301*** | -0.481*** | -0.232** | -0.391*** | -0.314** |
|  | [0.07] | [0.06] | [0.07] | [0.10] | [0.15] | [0.10] | [0.11] | [0.12] |
| Caste: ST | 0.066 | -0.116 | -0.233*** | -0.265** | -0.059 | -0.407*** | -0.314*** | -0.169 |
|  | [0.09] | [0.07] | [0.08] | [0.12] | [0.18] | [0.11] | [0.12] | [0.13] |
| Caste: BC | -0.147** | -0.099* | -0.091 | -0.265*** | -0.300** | -0.127 | -0.246*** | -0.244** |
|  | [0.06] | [0.05] | [0.06] | [0.10] | [0.13] | [0.08] | [0.09] | [0.11] |
| Religion: Hindu | -0.126 | -0.026 | -0.061 | 0.096 | -0.365* | 0.061 | 0.119 | 0.148 |
|  | [0.10] | [0.08] | [0.10] | [0.11] | [0.20] | [0.14] | [0.16] | [0.16] |
| Religion: Muslim | -0.381** | -0.123 | -0.249 | -0.447** | -0.848*** | -0.400 | -0.542* | -0.512* |
|  | [0.16] | [0.12] | [0.16] | [0.22] | [0.30] | [0.28] | [0.33] | [0.28] |
| Wealth: Poor | 0.111* | 0.038 | 0.070 | 0.158** | -0.033 | 0.124 | 0.216** | 0.057 |
|  | [0.06] | [0.05] | [0.05] | [0.08] | [0.12] | [0.09] | [0.10] | [0.09] |
| Wealth: Middle | 0.142** | 0.096* | 0.151*** | 0.180** | 0.163 | 0.266*** | 0.329*** | 0.255*** |
|  | [0.06] | [0.06] | [0.06] | [0.08] | [0.12] | [0.08] | [0.11] | [0.09] |
| Wealth: Rich | 0.107 | 0.123** | 0.187*** | 0.250** | 0.228* | 0.269*** | 0.345*** | 0.310*** |
|  | [0.07] | [0.06] | [0.06] | [0.11] | [0.14] | [0.09] | [0.11] | [0.10] |
| Wealth: Richest | 0.610*** | 0.310*** | 0.461*** | 0.441*** | 0.755*** | 0.672*** | 0.838*** | 0.809*** |
|  | [0.11] | [0.09] | [0.11] | [0.16] | [0.23] | [0.15] | [0.15] | [0.17] |
| Constant | -0.955* | -0.814** | -0.184 | 0.502 | 0.597 | -2.018*** | -2.246*** | -2.609*** |
|  | [0.50] | [0.41] | [0.45] | [0.91] | [1.02] | [0.75] | [0.87] | [0.79] |
| Observations | 1,264 | 1,264 | 1,264 | 1,264 | 1,264 | 1,264 | 1,264 | 1,264 |
| R-squared | 0.194 | 0.222 | 0.182 | 0.083 | 0.127 | 0.240 | 0.181 | 0.184 |
| District FE | Yes | Yes | Yes | Yes | Yes | Yes | Yes | Yes |

⁎⁎⁎ p < 0.01, ⁎⁎ p < 0.05, ⁎ p < 0.1.

Notes: Robust standard errors in brackets.

Table A2. OLS estimates of rainfall shock on educational outcomes - Results for all the covariates along with the main variable of interest in table 5.

|  | (1) | (2) | (3) | (4) | (5) |
| --- | --- | --- | --- | --- | --- |
|  | Completed Primary School | Completed Middle School | Completed Secondary School | Grade | STEM |
|  |  |  |  |  |  |
| Shock in birth year | -0.013* | -0.017* | -0.020** | -0.030 | -0.001 |
|  | [0.01] | [0.01] | [0.01] | [0.02] | [0.01] |
| Male | 0.142*** | 0.169*** | 0.142*** | -0.022 | 0.161*** |
|  | [0.01] | [0.01] | [0.01] | [0.02] | [0.02] |
| Household Head's Education | 0.020*** | 0.024*** | 0.019*** | 0.010*** | 0.006*** |
|  | [0.00] | [0.00] | [0.00] | [0.00] | [0.00] |
| Household Size | -0.003** | -0.005*** | -0.004*** | 0.002 | -0.002 |
|  | [0.00] | [0.00] | [0.00] | [0.00] | [0.00] |
| Caste: SC | -0.059*** | -0.080*** | -0.064*** | -0.096** | -0.009 |
|  | [0.02] | [0.02] | [0.02] | [0.04] | [0.02] |
| Caste: ST | -0.092*** | -0.097*** | -0.060** | -0.118** | -0.046 |
|  | [0.02] | [0.02] | [0.02] | [0.05] | [0.03] |
| Caste: BC | -0.047*** | -0.057*** | -0.038*** | -0.054* | -0.009 |
|  | [0.01] | [0.02] | [0.01] | [0.03] | [0.02] |
| Religion: Hindu | -0.028* | -0.048** | -0.007 | 0.068 | 0.038 |
|  | [0.02] | [0.02] | [0.03] | [0.04] | [0.03] |
| Religion: Muslim | -0.154*** | -0.235*** | -0.148*** | 0.046 | 0.091** |
|  | [0.02] | [0.03] | [0.04] | [0.06] | [0.04] |
| Wealth: Poor | 0.151*** | 0.176*** | 0.118*** | 0.021 | 0.026 |
|  | [0.01] | [0.01] | [0.01] | [0.03] | [0.02] |
| Wealth: Middle | 0.216*** | 0.280*** | 0.228*** | 0.060* | 0.026 |
|  | [0.01] | [0.02] | [0.02] | [0.03] | [0.02] |
| Wealth: Rich | 0.268*** | 0.350*** | 0.334*** | 0.116*** | 0.078*** |
|  | [0.01] | [0.02] | [0.02] | [0.04] | [0.02] |
| Wealth: Richest | 0.296*** | 0.433*** | 0.493*** | 0.201*** | 0.110*** |
|  | [0.02] | [0.02] | [0.03] | [0.05] | [0.03] |
| Constant | 0.598*** | 0.597*** | 0.469*** | 0.900*** | 0.046 |
|  | [0.04] | [0.05] | [0.04] | [0.09] | [0.06] |
|  |  |  |  |  |  |
| Observations | 35,926 | 31,464 | 28,478 | 9,044 | 6,817 |
| R-squared | 0.297 | 0.334 | 0.291 | 0.232 | 0.289 |
| District FE | Yes | Yes | Yes | Yes | Yes |
| Age FE | Yes | Yes | Yes | Yes | Yes |

⁎⁎⁎ p < 0.01, ⁎⁎ p < 0.05, ⁎ p < 0.1.

Notes: Robust standard errors in brackets are clustered at the district level.

Table A3. OLS estimates of rainfall shock on STEM including commerce.

|  | STEM |
| --- | --- |
|  |  |
| Shock in birth year | 0.009 |
|  | [0.02] |
|  |  |
| Observations | 6,817 |
| R-squared | 0.335 |
| Other Covariates | Yes |
| District FE | Yes |
| Age FE | Yes |

⁎⁎⁎ p < 0.01, ⁎⁎ p < 0.05, ⁎ p < 0.1.

Notes: Robust standard errors in brackets are clustered at the district level. “Other covariates” include the gender, household head's education, family size, religion, caste, and wealth status.

Table A4. OLS estimates of rainfall shock on grade in secondary school and STEM along with Lee bounds.

|  | (1) | (2) |
| --- | --- | --- |
|  | Grade | STEM |
| Shock in birth year |  |  |
|  |  |  |
| Overall Effect | -0.021 | -0.017* |
|  | [0.01] | [0.01] |
| Lee Bound: Lower | -0.045** | -0.022** |
|  | [0.02] | [0.01] |
| Lee Bound: Upper | 0.001 | 0.007 |
|  | [0.02] | [0.02] |
|  |  |  |
| Observations in overall sample | 21,213 | 21,362 |
| Observations in analytical sample | 9,044 | 6817 |
| Trimming Proportion | 0.02 | 0.03 |

Notes: Overall effect is estimated through OLS estimation of rainfall shock on test scores based on the naïve specification. Overall sample includes the number of observations with missing data for the outcome variable. Lower and upper bounds are estimated through Lee bounds estimation.

⁎⁎⁎ p < 0.01, ⁎⁎ p < 0.05, ⁎ p < 0.1.

Table A5. Summary Statistics of analytical and attrition sample.

| Variables | Analytical Sample | | Attrition Sample | | Difference |
| --- | --- | --- | --- | --- | --- |
|  | Mean | N | Mean | N |  |
| Male (=1 if yes) | 0.537 | 1264 | 0.494 | 239 | 0.044 |
| Father's Formal Education (=1 if yes) | 0.588 | 1264 | 0.420 | 205 | 0.168*** |
| Mother's Formal Education (=1 if yes) | 0.375 | 1264 | 0.201 | 204 | 0.174*** |
| Mother's Height (in cm) | 151.369 | 1264 | 150.540 | 178 | 0.830 |
| Household Size | 5.601 | 1264 | 5.623 | 239 | -0.022 |
| Caste |  |  |  |  |  |
| SC ( =1 if yes) | 0.206 | 1264 | 0.222 | 239 | -0.016 |
| ST ( =1 if yes) | 0.176 | 1264 | 0.209 | 239 | -0.033 |
| BC ( =1 if yes) | 0.474 | 1264 | 0.460 | 239 | 0.014 |
| OC ( =1 if yes) | 0.144 | 1264 | 0.109 | 239 | 0.035 |
| Religion |  |  |  |  |  |
| Hindu ( =1 if yes) | 0.918 | 1264 | 0.937 | 239 | -0.020 |
| Muslim ( =1 if yes) | 0.027 | 1264 | 0.013 | 239 | 0.014* |
| Others ( =1 if yes) | 0.055 | 1264 | 0.050 | 239 | 0.005 |
| Wealth Status |  |  |  |  |  |
| Poorest ( =1 if yes) | 0.249 | 1264 | 0.333 | 234 | -0.084** |
| Poor ( =1 if yes) | 0.269 | 1264 | 0.274 | 234 | -0.005 |
| Middle ( =1 if yes) | 0.251 | 1264 | 0.235 | 234 | 0.016 |
| Rich ( =1 if yes) | 0.187 | 1264 | 0.132 | 234 | 0.054** |
| Richest ( =1 if yes) | 0.044 | 1264 | 0.026 | 234 | 0.019 |
|  | | | | | |

Notes: Children included in the analysis are considered as the analytical sample and those children who could not be included in the analysis due to unavailability in remaining rounds, missing data in outcome variables and other covariates are considered as the attrition sample. *** p<0.01, ** p<0.05, * p<0.1

Table A6. Marginal effect of predictors of sample retention in the analytical sample.

| Variables | Inclusion in the analytical Sample |
| --- | --- |
| Male (=1 if yes) | 0.028* |
|  | [0.02] |
| Antenatal visit during pregnancy (=1 if yes) | 0.03 |
|  | [0.03] |
| Antenatal visit during pregnancy (=1 if not known or information not available) | -0.215** |
|  | [0.11] |
| Serious injury or illness (=1 if yes) | -0.009 |
|  | [0.02] |
| Number of children born to mother | -0.008 |
|  | [0.01] |
| Father's Formal Education (=1 if yes) | 0.045** |
|  | [0.02] |
| Mother's Formal Education (=1 if yes) | 0.045** |
|  | [0.02] |
| Caste: SC ( =1 if yes) | 0.007 |
|  | [0.03] |
| Caste: ST ( =1 if yes) | -0.004 |
|  | [0.03] |
| Caste: BC ( =1 if yes) | 0.003 |
|  | [0.03] |
| Religion: Hindu (=1 if yes) | -0.02 |
|  | [0.03] |
| Religion: Muslim (=1 if yes) | 0.095*** |
|  | [0.03] |
| Household Size | 0 |
|  | [0.00] |
| Wealth: Poorest | -0.023 |
|  | [0.06] |
| Wealth: Poor | 0.008 |
|  | [0.05] |
| Wealth: Middle | -0.002 |
|  | [0.06] |
| Wealth: Rich | 0.015 |
|  | [0.05] |
| District: Srikakulam | -0.023 |
|  | [0.04] |
| District: YSR | -0.123* |
|  | [0.07] |
| District: Ananth Puram | 0.032 |
|  | [0.04] |
| District: Karimnagar | 0.025 |
|  | [0.05] |
| District: Mahbubnagar | -0.071 |
|  | [0.05] |
| Observations | 1,462 |

Notes: Robust standard errors in brackets. Number of observations reduces from 1503 to 1462 due to perfect prediction within the following groups: not known or information not available for the father's (34 observations) and mother's education (2 observations) and no information on wealth status (6 observations). Outcome variable is a binary variable that takes a value one if the observation is include in the analytical sample and zero if the observations who could not be included in the analysis due to unavailability in remaining rounds, missing data in outcome variables and other covariates in the main analysis. *** p<0.01, ** p<0.05, * p<0.1.

Table A7. OLS estimates of rainfall shock on test scores after accounting for inverse probability weights.

|  | PPVT | | | | MAT | | | |
| --- | --- | --- | --- | --- | --- | --- | --- | --- |
|  | (1) | (2) | (3) | (4) | (5) | (6) | (7) | (8) |
|  | Age 5 | Age 8 | Age 12 | Age 15 | Age 5 | Age 8 | Age 12 | Age 15 |
| ***Panel A: Without School Enrollment Status*** | | | |  |  |  |  |  |
| Shock in birth year | -0.176*** | -0.080* | -0.052 | -0.042 | -0.384*** | -0.272*** | -0.09 | -0.017 |
|  | [0.05] | [0.05] | [0.05] | [0.07] | [0.10] | [0.07] | [0.09] | [0.08] |
|  |  |  |  |  |  |  |  |  |
| Observations | 1,264 | 1,264 | 1,264 | 1,264 | 1,264 | 1,264 | 1,264 | 1,264 |
| R-squared | 0.191 | 0.224 | 0.185 | 0.084 | 0.123 | 0.242 | 0.184 | 0.187 |
|  |  |  |  |  |  |  |  |  |
| ***Panel B: With School Enrollment Status*** | | | |  |  |  |  |  |
| Shock in birth year | -0.179*** | -0.064 | -0.051 | -0.056 | -0.411*** | -0.225*** | -0.084 | -0.044 |
|  | [0.05] | [0.04] | [0.05] | [0.07] | [0.10] | [0.07] | [0.09] | [0.07] |
|  |  |  |  |  |  |  |  |  |
| Observations | 1,264 | 1,264 | 1,264 | 1,264 | 1,264 | 1,264 | 1,264 | 1,264 |
| R-squared | 0.191 | 0.234 | 0.186 | 0.12 | 0.126 | 0.271 | 0.19 | 0.281 |
| Other Covariates | Yes | Yes | Yes | Yes | Yes | Yes | Yes | Yes |
| District FE | Yes | Yes | Yes | Yes | Yes | Yes | Yes | Yes |

Notes: Robust standard errors in brackets. Inverse probability weights are used as the probability weights. “Other covariates” include the gender of the child, father's education, mother's education, mother's height, family size, religion, caste, and wealth status. In addition to these covariates, Panel B also includes an indicator variable equal to 1 for children enrolled in pre-school/school at the age when the test was conducted and 0 otherwise.
⁎⁎⁎ p < 0.01, ⁎⁎ p < 0.05, ⁎ p < 0.1.

Table A8. OLS estimates of rainfall shock on test scores after controlling for test scores at the age of 5 years and inverse probability weights.

|  | PPVT | | | MAT | | |
| --- | --- | --- | --- | --- | --- | --- |
|  | (1) | (2) | (3) | (4) | (5) | (6) |
|  | Age 8 | Age 12 | Age 15 | Age 8 | Age 12 | Age 15 |
| ***Panel A: Without School Enrollment Status*** | | | |  |  |  |
| Shock in birth year | -0.036 | -0.011 | -0.016 | -0.195*** | -0.021 | 0.045 |
|  | [0.04] | [0.05] | [0.07] | [0.07] | [0.09] | [0.08] |
| PPVT score (Age 5) | 0.250*** | 0.234*** | 0.147*** |  |  |  |
|  | [0.03] | [0.03] | [0.04] |  |  |  |
| MAT score (Age 5) | |  |  | 0.200*** | 0.179*** | 0.160*** |
|  |  |  |  | [0.02] | [0.02] | [0.02] |
|  |  |  |  |  |  |  |
| Observations | 1,264 | 1,264 | 1,264 | 1,264 | 1,264 | 1,264 |
| R-squared | 0.289 | 0.236 | 0.094 | 0.298 | 0.22 | 0.219 |
|  |  |  |  |  |  |  |
| ***Panel B: With School Enrollment Status*** | | | |  |  |  |
| Shock in birth year | -0.021 | -0.009 | -0.032 | -0.148** | -0.016 | 0.013 |
|  | [0.04] | [0.05] | [0.07] | [0.07] | [0.09] | [0.08] |
| PPVT score (Age 5) | 0.248*** | 0.234*** | 0.138*** |  |  |  |
|  | [0.03] | [0.03] | [0.04] |  |  |  |
| Math score (Age 5) | |  |  | 0.200*** | 0.177*** | 0.145*** |
|  |  |  |  | [0.02] | [0.02] | [0.02] |
|  |  |  |  |  |  |  |
| Enrolled | 0.824 | 0.367*** | 0.680*** | 2.528*** | 1.126* | 1.297*** |
|  | [0.55] | [0.12] | [0.11] | [0.78] | [0.62] | [0.16] |
|  |  |  |  |  |  |  |
| Observations | 1,264 | 1,264 | 1,264 | 1,264 | 1,264 | 1,264 |
| R-squared | 0.298 | 0.238 | 0.129 | 0.329 | 0.225 | 0.307 |
| Other Covariates | Yes | Yes | Yes | Yes | Yes | Yes |
| District FE | Yes | Yes | Yes | Yes | Yes | Yes |

Notes: Robust standard errors in brackets. Inverse probability weights are used as the probability weights. “Other covariates” include the gender of the child, father's education, mother's education, mother's height, family size, religion, caste, and wealth status. In addition to these covariates, Panel B also includes an indicator variable equal to 1 for children enrolled in pre-school/school at the age when the test was conducted and 0 otherwise.
⁎⁎⁎ p < 0.01, ⁎⁎ p < 0.05, ⁎ p < 0.1.

Table A9. OLS estimates of rainfall shocks on test scores.

|  | PPVT | | | | MAT | | | |
| --- | --- | --- | --- | --- | --- | --- | --- | --- |
|  | (1) | (2) | (3) | (4) | (5) | (6) | (7) | (8) |
|  | Age 5 | Age 8 | Age 12 | Age 15 | Age 5 | Age 8 | Age 12 | Age 15 |
| ***Panel A: Without School Enrollment Status*** | | | |  |  |  |  |  |
| Shock in birth year | -0.224*** | -0.145** | -0.078 | -0.104 | -0.285* | -0.294** | -0.257 | -0.232* |
|  | [0.07] | [0.06] | [0.07] | [0.09] | [0.16] | [0.14] | [0.17] | [0.14] |
| Shock at age 1 | -0.134 | -0.195 | -0.079 | -0.172 | 0.283 | -0.069 | -0.527 | -0.631* |
|  | [0.19] | [0.17] | [0.18] | [0.26] | [0.40] | [0.32] | [0.40] | [0.34] |
| Shock at age 2 | -0.079 | -0.172* | -0.086 | -0.171 | 0.145 | -0.101 | -0.382* | -0.553*** |
|  | [0.10] | [0.10] | [0.12] | [0.16] | [0.22] | [0.17] | [0.23] | [0.20] |
|  |  |  |  |  |  |  |  |  |
| Observations | 1,264 | 1,264 | 1,264 | 1,264 | 1,264 | 1,264 | 1,264 | 1,264 |
| R-squared | 0.188 | 0.223 | 0.183 | 0.083 | 0.125 | 0.239 | 0.182 | 0.189 |
| ***Panel B: With School Enrollment Status*** | | |  |  |  |  |  |  |
| Shock in birth year | -0.224*** | -0.127* | -0.076 | -0.05 | -0.303* | -0.239* | -0.252 | -0.128 |
|  | [0.07] | [0.06] | [0.07] | [0.09] | [0.16] | [0.13] | [0.17] | [0.13] |
| Shock at age 1 | -0.133 | -0.182 | -0.08 | 0.024 | 0.308 | -0.031 | -0.528 | -0.249 |
|  | [0.19] | [0.18] | [0.18] | [0.25] | [0.40] | [0.31] | [0.39] | [0.31] |
| Shock at age 2 | -0.077 | -0.161 | -0.086 | -0.018 | 0.178 | -0.066 | -0.382* | -0.255 |
|  | [0.10] | [0.10] | [0.12] | [0.16] | [0.22] | [0.17] | [0.23] | [0.18] |
|  |  |  |  |  |  |  |  |  |
| Observations | 1,264 | 1,264 | 1,264 | 1,264 | 1,264 | 1,264 | 1,264 | 1,264 |
| R-squared | 0.188 | 0.232 | 0.184 | 0.117 | 0.128 | 0.266 | 0.187 | 0.279 |
| Other Covariates | Yes | Yes | Yes | Yes | Yes | Yes | Yes | Yes |
| District FE | Yes | Yes | Yes | Yes | Yes | Yes | Yes | Yes |

Notes: Robust standard errors in brackets. “Other covariates” include the gender of the child, father's education, mother's education, mother's height, family size, religion, caste, and wealth status. In addition to these covariates, Panel B also includes an indicator variable equal to 1 for children enrolled in pre-school/school at the age when the test was conducted and 0 otherwise.

⁎⁎⁎ p < 0.01, ⁎⁎ p < 0.05, ⁎ p < 0.1.

Table A10. OLS estimates of rainfall shocks on test scores after controlling for test scores at the age of 5 years.

|  | PPVT | | | | | | MAT | | | | |
| --- | --- | --- | --- | --- | --- | --- | --- | --- | --- | --- | --- |
|  | (1) | | (2) | | (3) | | (4) | | (5) | | (6) |
|  | Age 8 | | Age 12 | | Age 15 | | Age 8 | | Age 12 | | Age 15 |
| ***Panel A: Without School Enrollment Status*** | | | | | |  | |  | |  | |
| Shock in birth year | -0.089 | | -0.026 | | -0.072 | | -0.236* | | -0.205 | | -0.185 |
|  | [0.06] | | [0.07] | | [0.09] | | [0.13] | | [0.17] | | [0.14] |
| Shock at age 1 | -0.161 | | -0.048 | | -0.153 | | -0.127 | | -0.578 | | -0.679** |
|  | [0.17] | | [0.18] | | [0.26] | | [0.32] | | [0.39] | | [0.34] |
| Shock at age 2 | -0.153 | | -0.068 | | -0.16 | | -0.13 | | -0.409* | | -0.577*** |
|  | [0.10] | | [0.12] | | [0.16] | | [0.17] | | [0.22] | | [0.20] |
| PPVT score (Age 5) | 0.251*** | | 0.231*** | | 0.145*** | |  | |  | |  |
|  | [0.03] | | [0.03] | | [0.04] | |  | |  | |  |
| MAT score (Age 5) | |  | |  | | 0.205*** | | 0.181*** | | 0.168*** | |
|  |  | |  | |  | | [0.02] | | [0.02] | | [0.02] |
|  |  | |  | |  | |  | |  | |  |
| Observations | 1,264 | | 1,264 | | 1,264 | | 1,264 | | 1,264 | | 1,264 |
| R-squared | 0.289 | | 0.234 | | 0.092 | | 0.299 | | 0.22 | | 0.225 |
| ***Panel B: With School Enrollment Status*** | | | | | |  | |  | |  | |
| Shock in birth year | -0.072 | | -0.025 | | -0.021 | | -0.181 | | -0.201 | | -0.089 |
|  | [0.06] | | [0.07] | | [0.09] | | [0.13] | | [0.17] | | [0.13] |
| Shock at age 1 | -0.149 | | -0.049 | | 0.039 | | -0.089 | | -0.579 | | -0.305 |
|  | [0.17] | | [0.18] | | [0.25] | | [0.30] | | [0.39] | | [0.31] |
| Shock at age 2 | -0.141 | | -0.068 | | -0.01 | | -0.095 | | -0.408* | | -0.287 |
|  | [0.10] | | [0.12] | | [0.16] | | [0.16] | | [0.22] | | [0.18] |
| PPVT score (Age 5) | 0.250*** | | 0.231*** | | 0.136*** | |  | |  | |  |
|  | [0.03] | | [0.03] | | [0.04] | |  | |  | |  |
| Math score (Age 5) | |  | |  | | 0.205*** | | 0.179*** | | 0.152*** | |
|  |  | |  | |  | | [0.02] | | [0.02] | | [0.02] |
|  |  | |  | |  | |  | |  | |  |
| Enrolled | 0.795 | | 0.367*** | | 0.678*** | | 2.471*** | | 1.160* | | 1.292*** |
|  | [0.54] | | [0.12] | | [0.12] | | [0.75] | | [0.62] | | [0.16] |
| Observations | 1,264 | | 1,264 | | 1,264 | | 1,264 | | 1,264 | | 1,264 |
| R-squared | 0.297 | | 0.235 | | 0.126 | | 0.326 | | 0.224 | | 0.308 |

Notes: Robust standard errors in brackets. “Other covariates” include the gender of the child, father's education, mother's education, mother's height, family size, religion, caste, and wealth status. In addition to these covariates, Panel B also includes an indicator variable equal to 1 for children enrolled in pre-school/school at the age when the test was conducted and 0 otherwise.

⁎⁎⁎ p < 0.01, ⁎⁎ p < 0.05, ⁎ p < 0.1.

Table A11. OLS estimates of rainfall shocks on educational outcomes.

|  | (1) | (2) | (3) | (4) | (5) |
| --- | --- | --- | --- | --- | --- |
|  | Completed Primary School | Completed Middle School | Completed Secondary School | Grade | STEM |
| Shock in birth year | -0.012* | -0.017* | -0.018* | -0.029 | 0.000 |
|  | [0.01] | [0.01] | [0.01] | [0.02] | [0.01] |
| Shock at age 1 | -0.004 | -0.001 | -0.016* | 0.008 | -0.007 |
|  | [0.01] | [0.01] | [0.01] | [0.02] | [0.01] |
| Shock at age 2 | -0.01 | -0.006 | -0.007 | -0.046** | 0.005 |
|  | [0.01] | [0.01] | [0.01] | [0.02] | [0.02] |
|  |  |  |  |  |  |
| Observations | 35,926 | 31,464 | 28,478 | 9,044 | 6,817 |
| R-squared | 0.298 | 0.334 | 0.291 | 0.233 | 0.289 |
| Other Covariates | Yes | Yes | Yes | Yes | Yes |
| District FE | Yes | Yes | Yes | Yes | Yes |
| Age FE | Yes | Yes | Yes | Yes | Yes |

Notes: Robust standard errors in brackets are clustered at the district level. “Other covariates” include the gender, household head's education, family size, religion, caste, and wealth status.

⁎⁎⁎ p < 0.01, ⁎⁎ p < 0.05, ⁎ p < 0.1.

Table A12. OLS estimates of extreme rainfall shock on test scores.

|  | PPVT | | | | MAT | | | |
| --- | --- | --- | --- | --- | --- | --- | --- | --- |
|  | (1) | (2) | (3) | (4) | (5) | (6) | (7) | (8) |
|  | Age 5 | Age 8 | Age 12 | Age 15 | Age 5 | Age 8 | Age 12 | Age 15 |
| ***Panel A: Without School Enrollment Status*** | | | |  |  |  |  |  |
| Shock in birth year | -0.211*** | -0.089** | -0.058 | -0.053 | -0.405*** | -0.282*** | -0.083 | -0.004 |
|  | [0.05] | [0.04] | [0.04] | [0.07] | [0.09] | [0.07] | [0.08] | [0.07] |
|  |  |  |  |  |  |  |  |  |
| Observations | 1,264 | 1,264 | 1,264 | 1,264 | 1,264 | 1,264 | 1,264 | 1,264 |
| R-squared | 0.192 | 0.222 | 0.183 | 0.082 | 0.127 | 0.241 | 0.18 | 0.182 |
| ***Panel B: With School Enrollment Status*** | | | |  |  |  |  |  |
| Shock in birth year | -0.214*** | -0.077* | -0.057 | -0.072 | -0.435*** | -0.246*** | -0.079 | -0.042 |
|  | [0.05] | [0.04] | [0.04] | [0.06] | [0.09] | [0.07] | [0.08] | [0.07] |
|  |  |  |  |  |  |  |  |  |
| Observations | 1,264 | 1,264 | 1,264 | 1,264 | 1,264 | 1,264 | 1,264 | 1,264 |
| R-squared | 0.192 | 0.231 | 0.184 | 0.117 | 0.13 | 0.268 | 0.185 | 0.277 |
| Other Covariates | Yes | Yes | Yes | Yes | Yes | Yes | Yes | Yes |
| District FE | Yes | Yes | Yes | Yes | Yes | Yes | Yes | Yes |

Notes: Robust standard errors in brackets. “Other covariates” include the gender of the child, father's education, mother's education, mother's height, family size, religion, caste, and wealth status. In addition to these covariates, Panel B also includes an indicator variable equal to 1 for children enrolled in pre-school/school at the age when the test was conducted and 0 otherwise. Extreme rainfall shocks are constructed using Shah and Steinberg (2017) strategy.

⁎⁎⁎ p < 0.01, ⁎⁎ p < 0.05, ⁎ p < 0.1.

Table A13. OLS estimates of extreme rainfall shock on test scores after controlling for test scores at the age of 5 years.

|  | PPVT | | | MAT | | | |
| --- | --- | --- | --- | --- | --- | --- | --- |
|  | (1) | (2) | (3) | (4) | (5) | (6) |  |
|  | Age 8 | Age 12 | Age 15 | Age 8 | Age 12 | Age 15 |  |
| ***Panel A: Without School Enrollment Status*** | | | |  |  |  |  |
| Shock in birth year | -0.036 | -0.009 | -0.022 | -0.199*** | -0.01 | 0.064 |  |
|  | [0.04] | [0.04] | [0.07] | [0.07] | [0.08] | [0.08] |  |
| PPVT score (Age 5) | 0.251*** | 0.230*** | 0.144*** |  |  |  |  |
|  | [0.03] | [0.03] | [0.04] |  |  |  |  |
| MAT score (Age 5) | |  |  | 0.203*** | 0.180*** | 0.167*** |  |
|  |  |  |  | [0.02] | [0.02] | [0.02] |  |
|  |  |  |  |  |  |  |  |
| Observations | 1,264 | 1,264 | 1,264 | 1,264 | 1,264 | 1,264 |  |
| R-squared | 0.288 | 0.233 | 0.091 | 0.3 | 0.217 | 0.217 |  |
|  |  |  |  |  |  |  |  |
| ***Panel B: With School Enrollment Status*** | | | |  |  |  |  |
| Shock in birth year | -0.025 | -0.008 | -0.043 | -0.164** | -0.007 | 0.021 |  |
|  | [0.04] | [0.04] | [0.07] | [0.07] | [0.08] | [0.07] |  |
| PPVT score (Age 5) | 0.249*** | 0.231*** | 0.135*** |  |  |  |  |
|  | [0.03] | [0.03] | [0.04] |  |  |  |  |
| Math score (Age 5) | |  |  | 0.203*** | 0.178*** | 0.151*** |  |
|  |  |  |  | [0.02] | [0.02] | [0.02] |  |
| Enrolled | 0.804 | 0.367*** | 0.681*** | 2.476*** | 1.160* | 1.320*** |  |
|  | [0.53] | [0.12] | [0.11] | [0.75] | [0.63] | [0.16] |  |
|  |  |  |  |  |  |  |  |
| Observations | 1,264 | 1,264 | 1,264 | 1,264 | 1,264 | 1,264 |  |
| R-squared | 0.296 | 0.235 | 0.126 | 0.327 | 0.222 | 0.306 |  |
| Other Covariates | Yes | Yes | Yes | Yes | Yes | Yes |  |
| District FE | Yes | Yes | Yes | Yes | Yes | Yes |  |

Notes: Robust standard errors in brackets. “Other covariates” include the gender of the child, father's education, mother's education, mother's height, family size, religion, caste, and wealth status. In addition to these covariates, Panel B also includes an indicator variable equal to 1 for children enrolled in pre-school/school at the age when the test was conducted and 0 otherwise. Extreme rainfall shocks are constructed using Shah and Steinberg (2017) strategy.

⁎⁎⁎ p < 0.01, ⁎⁎ p < 0.05, ⁎ p < 0.1.

Table A14. OLS estimates of extreme rainfall shock on educational outcomes.

|  | (1) | (2) | (3) | (4) | (5) |
| --- | --- | --- | --- | --- | --- |
|  | Completed Primary School | Completed Middle School | Completed Secondary School | Grade | STEM |
|  |  |  |  |  |  |
| Shock in birth year | 0.008 | 0.001 | -0.016 | -0.041* | 0.000 |
|  | [0.01] | [0.01] | [0.01] | [0.02] | [0.02] |
|  |  |  |  |  |  |
| Observations | 35,926 | 31,464 | 28,478 | 9,044 | 6,817 |
| R-squared | 0.297 | 0.334 | 0.29 | 0.232 | 0.289 |
| Other Covariates | Yes | Yes | Yes | Yes | Yes |
| District FE | Yes | Yes | Yes | Yes | Yes |
| Age FE | Yes | Yes | Yes | Yes | Yes |

Notes: Robust standard errors in brackets are clustered at the district level. “Other covariates” include the gender, household head's education, family size, religion, caste, and wealth status. Extreme rainfall shocks are constructed using Shah and Steinberg (2017) strategy.

⁎⁎⁎ p < 0.01, ⁎⁎ p < 0.05, ⁎ p < 0.1.

Table A15. OLS estimates of rainfall shock on test scores with standard errors clustered as the mandal level.

|  | (1) | (2) | (3) | (4) | (5) | | (6) | (7) | (8) |  |
| --- | --- | --- | --- | --- | --- | --- | --- | --- | --- | --- |
|  | PPVT | | | | | MAT | | | | |
|  | Age 5 | Age 8 | Age 12 | Age 15 | Age 5 | | Age 8 | Age 12 | Age 15 |  |
| ***Panel A: Without School Enrollment Status*** | | | |  |  | |  |  |  |  |
| Shock in birth year | -0.180*** | -0.074 | -0.049 | -0.041 | -0.385*** | | -0.269*** | -0.072 | -0.001 |  |
|  | [0.04] | [0.05] | [0.03] | [0.07] | [0.09] | | [0.06] | [0.10] | [0.10] |  |
|  |  |  |  |  |  | |  |  |  |  |
| Observations | 1,264 | 1,264 | 1,264 | 1,264 | 1,264 | | 1,264 | 1,264 | 1,264 |  |
| R-squared | 0.194 | 0.222 | 0.182 | 0.083 | 0.127 | | 0.240 | 0.181 | 0.184 |  |
|  |  |  |  |  |  | |  |  |  |  |
| ***Panel B: With School Enrollment Status*** | | | |  |  | |  |  |  |  |
| Shock in birth year | -0.182*** | -0.060 | -0.048 | -0.058 | -0.412*** | | -0.228*** | -0.066 | -0.036 |  |
|  | [0.04] | [0.05] | [0.03] | [0.07] | [0.09] | | [0.06] | [0.10] | [0.06] |  |
|  |  |  |  |  |  | |  |  |  |  |
| Observations | 1,264 | 1,264 | 1,264 | 1,264 | 1,264 | | 1,264 | 1,264 | 1,264 |  |
| R-squared | 0.194 | 0.231 | 0.184 | 0.118 | 0.130 | | 0.267 | 0.186 | 0.280 |  |
|  |  |  |  |  |  | |  |  |  |  |
| Other Covariates | Yes | Yes | Yes | Yes | Yes | | Yes | Yes | Yes |  |
| District FE | Yes | Yes | Yes | Yes | Yes | | Yes | Yes | Yes |  |

Notes: Robust standard errors in brackets are clustered at the mandal level. “Other covariates” include the gender of the child, father's education, mother's education, mother's height, family size, religion, caste, and wealth status. In addition to these covariates, Panel B also includes an indicator variable equal to 1 for children enrolled in pre-school/school at the age when the test was conducted and 0 otherwise.

⁎⁎⁎ p < 0.01, ⁎⁎ p < 0.05, ⁎ p < 0.1.

Table A16. OLS estimates of rainfall shock on test scores after controlling for test scores at the age of 5 years with standard errors clustered as the mandal level.

|  | (1) | (2) | (3) | (4) | (5) | (6) |
| --- | --- | --- | --- | --- | --- | --- |
|  | PPVT | | | MAT | | |
|  | Age 8 | Age 12 | Age 15 | Age 8 | Age 12 | Age 15 |
| ***Panel A: Without School Enrollment Status*** | | | |  |  |  |
| Shock in birth year | -0.029 | -0.008 | -0.015 | -0.191*** | -0.003 | 0.062 |
|  | [0.05] | [0.03] | [0.08] | [0.06] | [0.09] | [0.11] |
| PPVT score (Age 5) | 0.251*** | 0.230*** | 0.143** |  |  |  |
|  | [0.04] | [0.04] | [0.05] |  |  |  |
| MAT score (Age 5) | |  |  | 0.203*** | 0.179*** | 0.165*** |
|  |  |  |  | [0.02] | [0.02] | [0.03] |
|  |  |  |  |  |  |  |
| Observations | 1,264 | 1,264 | 1,264 | 1,264 | 1,264 | 1,264 |
| R-squared | 0.287 | 0.233 | 0.092 | 0.299 | 0.217 | 0.219 |
|  |  |  |  |  |  |  |
| ***Panel B: With School Enrollment Status*** | | | |  |  |  |
| Shock in birth year | -0.016 | -0.006 | -0.034 | -0.150** | 0.002 | 0.023 |
|  | [0.05] | [0.03] | [0.07] | [0.06] | [0.09] | [0.06] |
| PPVT score (Age 5) | 0.249*** | 0.230*** | 0.133** |  |  |  |
|  | [0.04] | [0.04] | [0.05] |  |  |  |
| Math score (Age 5) | |  |  | 0.203*** | 0.177*** | 0.149*** |
|  |  |  |  | [0.02] | [0.02] | [0.02] |
| Enrolled | 0.809 | 0.371*** | 0.681*** | 2.478*** | 1.160* | 1.322*** |
|  | [0.58] | [0.11] | [0.06] | [0.47] | [0.64] | [0.19] |
|  |  |  |  |  |  |  |
| Observations | 1,264 | 1,264 | 1,264 | 1,264 | 1,264 | 1,264 |
| R-squared | 0.296 | 0.234 | 0.126 | 0.326 | 0.222 | 0.307 |
|  |  |  |  |  |  |  |
| Other Covariates | Yes | Yes | Yes | Yes | Yes | Yes |
| District FE | Yes | Yes | Yes | Yes | Yes | Yes |

Notes: Robust standard errors in brackets are clustered at the mandal level. “Other covariates” include the gender of the child, father's education, mother's education, mother's height, family size, religion, caste, and wealth status. In addition to these covariates, Panel B also includes an indicator variable equal to 1 for children enrolled in pre-school/school at the age when the test was conducted and 0 otherwise.

⁎⁎⁎ p < 0.01, ⁎⁎ p < 0.05, ⁎ p < 0.1.

Table A17: OLS estimates of extreme rainfall shocks (alternative measure) and its interaction with gender on test scores and educational outcomes.

|  | (1) | (2) | (3) | (4) | (5) | (6) | (7) | (8) |
| --- | --- | --- | --- | --- | --- | --- | --- | --- |
| ***Panel A: YLS*** | PPVT | | | | MAT | | | |
|  | Age 5 | Age 8 | Age 12 | Age 15 | Age 5 | Age 8 | Age 12 | Age 15 |
| Shock in birth year*Male | -0.103 | -0.011 | -0.106 | -0.218 | -0.242 | -0.001 | 0.084 | -0.087 |
|  | [0.09] | [0.08] | [0.09] | [0.14] | [0.18] | [0.14] | [0.16] | [0.15] |
| Shock in birth year | -0.154** | -0.083 | 0.001 | 0.068 | -0.271** | -0.281*** | -0.129 | 0.044 |
|  | [0.07] | [0.06] | [0.06] | [0.11] | [0.13] | [0.10] | [0.12] | [0.11] |
| Male | 0.087* | 0.156*** | 0.172*** | 0.189*** | 0.148 | 0.089 | 0.128* | 0.313*** |
|  | [0.05] | [0.04] | [0.04] | [0.07] | [0.10] | [0.07] | [0.08] | [0.08] |
| Observations | 1,264 | 1,264 | 1,264 | 1,264 | 1,264 | 1,264 | 1,264 | 1,264 |
| R-squared | 0.193 | 0.222 | 0.184 | 0.084 | 0.129 | 0.241 | 0.180 | 0.183 |
| Other Covariates | Yes | Yes | Yes | Yes | Yes | Yes | Yes | Yes |
| ***Panel B: IHDS- Level Completion*** | Primary | Middle | Secondary | Grade | STEM |  |  |  |
| Shock in birth year*Male | -0.040** | -0.025 | -0.032 | 0.015 | -0.020 |  |  |  |
|  | [0.02] | [0.02] | [0.02] | [0.04] | [0.03] |  |  |  |
| Shock in birth year | 0.027** | 0.012 | 0.001 | -0.049 | 0.000 |  |  |  |
|  | [0.01] | [0.01] | [0.02] | [0.03] | [0.02] |  |  |  |
| Male | 0.149*** | 0.174*** | 0.148*** | -0.026 | 0.164*** |  |  |  |
|  | [0.01] | [0.01] | [0.01] | [0.02] | [0.02] |  |  |  |
| Observations | 35,926 | 31,464 | 28,478 | 9,044 | 6,817 |  |  |  |
| R-squared | 0.298 | 0.334 | 0.291 | 0.232 | 0.289 |  |  |  |
| Other Covariates | Yes | Yes | Yes | Yes | Yes |  |  |  |
| Age FE | Yes | Yes | Yes | Yes | Yes |  |  |  |

⁎⁎⁎ p < 0.01, ⁎⁎ p < 0.05, ⁎ p < 0.1.

Notes: Extreme rainfall shocks are constructed using followed Shah and Steinberg (2017) strategy. In Panel A, robust standard errors are reported in brackets and “Other covariates” include the father's education, mother's education, mother's height, family size, religion, caste, and wealth status. In Panel B, robust standard errors in brackets are clustered at the district level and “Other covariates” include the household head's education, family size, religion, caste, and wealth status. In both the panels, male is a binary variable indicating whether the child belongs to male cohort (=1) or female cohort (=0). District fixed effects are included in both the panels.

Table A18: Falsification Test: OLS estimates of extreme rainfall shock (alternative measure) in false birth year.

|  | (1) | (2) | (3) | (4) | (5) | (6) | (7) | (8) |
| --- | --- | --- | --- | --- | --- | --- | --- | --- |
| ***Panel A: YLS*** | PPVT | | | | MAT | | | |
|  | Age 5 | Age 8 | Age 12 | Age 15 | Age 5 | Age 8 | Age 12 | Age 15 |
|  |  |  |  |  |  |  |  |  |
| Shock in five years before birth | -0.029 | -0.012 | 0.003 | -0.001 | 0.066 | 0.014 | -0.073 | -0.040 |
|  | [0.06] | [0.06] | [0.05] | [0.07] | [0.13] | [0.10] | [0.10] | [0.10] |
|  |  |  |  |  |  |  |  |  |
| Observations | 1,264 | 1,264 | 1,264 | 1,264 | 1,264 | 1,264 | 1,264 | 1,264 |
| R-squared | 0.179 | 0.219 | 0.182 | 0.081 | 0.114 | 0.230 | 0.179 | 0.182 |
|  |  |  |  |  |  |  |  |  |
| Other Covariates | Yes | Yes | Yes | Yes | Yes | Yes | Yes | Yes |
| District FE | Yes | Yes | Yes | Yes | Yes | Yes | Yes | Yes |
|  | | | | | | | | |
| ***Panel B: IHDS*** | Completed  Primary  School | Completed  Middle  School | Completed  Secondary  School | Grade | STEM |  |  |  |
|  |  |  |  |  |  |  |  |  |
| Shock in five years before birth | -0.009 | -0.010 | -0.001 | 0.071*** | 0.015 |  |  |  |
|  | [0.01] | [0.01] | [0.01] | [0.02] | [0.02] |  |  |  |
|  |  |  |  |  |  |  |  |  |
| Observations | 35,926 | 31,464 | 28,478 | 9,044 | 6,817 |  |  |  |
| R-squared | 0.297 | 0.334 | 0.290 | 0.233 | 0.289 |  |  |  |
|  |  |  |  |  |  |  |  |  |
| Other Covariates | Yes | Yes | Yes | Yes | Yes |  |  |  |
| District FE | Yes | Yes | Yes | Yes | Yes |  |  |  |
| Age FE | Yes | Yes | Yes | Yes | Yes |  |  |  |

⁎⁎⁎ p < 0.01, ⁎⁎ p < 0.05, ⁎ p < 0.1.

Notes: Extreme rainfall shocks are constructed using followed Shah and Steinberg (2017) strategy. In Panel A, robust standard errors are reported in brackets and “Other covariates” include the gender of the child, father's education, mother's education, mother's height, family size, religion, caste, and wealth status. In Panel B, robust standard errors in brackets are clustered at the district level and “Other covariates” include the gender, household head's education, family size, religion, caste, and wealth status.Table A19. OLS estimates of extreme rainfall shock (alternative measure) on grade in secondary school and STEM along with Lee bounds.

|  | (1) | (2) |
| --- | --- | --- |
|  | Grade | STEM |
| Shock in birth year |  |  |
|  |  |  |
| Overall Effect | -0.052*** | -0.063*** |
|  | [0.02] | [0.01] |
| Lee Bound: Lower | -0.121*** | -0.088*** |
|  | [0.03] | [0.01] |
| Lee Bound: Upper | 0.010 | 0.035 |
|  | [0.03] | [0.03] |
|  |  |  |
| Observations in overall sample | 21,213 | 21,362 |
| Observations in analytical sample | 9,044 | 6817 |
| Trimming Proportion | 0.06 | 0.11 |

Notes: Overall effect is estimated through OLS estimation of extreme rainfall shock on test scores based on the naïve specification. Overall sample includes the number of observations with missing data for the outcome variable. Lower and upper bounds are estimated through Lee bounds estimation.

⁎⁎⁎ p < 0.01, ⁎⁎ p < 0.05, ⁎ p < 0.1.

**Appendix section B**

B1. The following statistics helps in understanding the correspondence between the original grid (latitude-longitude) level distance and the actual distance covered. A distance by the 0.5 degree of latitude is approximately same (55 km) across different points of longitude. However, distance covered by 0.5 degree of longitude varies by the point of latitude. For instance, the latitude of India ranges from 8°4’N and 37°6’N. The distance covered by 0.5 degree of longitude is approximately 55 km at 8°4’N and 44 km at 37°6’N. Hence, a 0.5° × 0.5° grid will be (approximately) between 55 km by 55 km (at 8°4’N) and 44 km by 55 km (at 37°6’N).

B2. Cognitive Development assessment at Age 5 (CDA): This test requires basic knowledge of quantitative skills. We consider it as a basic mathematics test for this analysis.

B3: In Indian school system, at the higher secondary level, usually students need to opt a particular stream of subjects, like Arts, Science, Commerce etc. which are mostly irreversible, that is, once a stream is selected at the higher secondary school, students cannot change the stream later in their college. Although studying literature in college is an exception, because students from any stream has to mandatorily study that till high school.

B4: This is a finding from a naïve OLS estimation, with dependent variable being the binary choice of STEM versus other subjects, and the binary predictor variable being receiving 1^st^ division grade in secondary examination versus receiving any other passing grades. Since there are no other variables in this model, we do not report it separately in the text. This has been done to get an idea about the strengths of association between these two binary variables.
